# Non-Fragile Estimation for Nonlinear Delayed Complex Networks with Random Couplings Using Binary Encoding Schemes

**DOI:** 10.3390/s25092880

**Published:** 2025-05-02

**Authors:** Nan Hou, Weijian Li, Yanhua Song, Mengdi Chang, Xianye Bu

**Affiliations:** 1 Sanya Offshore Oil & Gas Research Institute, Northeast Petroleum University, Sanya 572025, China; nanhou@nepu.edu.cn; 2 Artificial Intelligence Energy Research Institute, Northeast Petroleum University, Daqing 163318, China; hytsyh@126.com (Y.S.); cmd_1230@163.com (M.C.); 3 National Key Laboratory of Continental Shale Oil, Northeast Petroleum University, Daqing 163318, China; 4 Heilongjiang Provincial Key Laboratory of Networking and Intelligent Control, Northeast Petroleum University, Daqing 163318, China; 5 Research Center for Mathematics and Interdisciplinary Sciences, Northeast Petroleum University, Daqing 163318, China; 6 Sinopec Qilu Petrochemical Company, Zibo 255400, China; lwjyeokrin@163.com; 7 School of Electrical & Information Engineering, Northeast Petroleum University, Daqing 163318, China

**Keywords:** complex network, binary encoding schemes, random couplings, state estimation, randomly occurring multiple delays

## Abstract

This paper is dedicated to dealing with the design issue of a non-fragile state estimator for a type of nonlinear complex network subject to random couplings and random multiple time delays under binary encoding schemes (BESs). The BESs are put into use in the transmission of data from the sensor to the remote estimator. The phenomenon of bit errors is considered in the process of signal transmission, whose description utilizes a Bernoulli-distributed random sequence. The random couplings are represented by using the Kronecker delta function as well as a Markov chain. This paper aims to conduct a non-fragile state estimation such that, in the presence of some variations/perturbations in the gain parameter of the estimator, the estimation error dynamics will reach exponential ultimate boundedness in mean square and the ultimate bound will be minimized. Utilizing both stochastic analysis and matrix inequality processing, a sufficient condition is provided to guarantee that the constructed estimator satisfies the expected estimation performance, and the estimator gains are acquired by tackling an optimization issue constrained by some linear matrix inequalities. Eventually, two simulation examples are conducted, whose results verify that the approach to the design of a non-fragile estimator in this paper is effective.

## 1. Introduction

Complex networks (CNs) are large-scale networked systems comprising numerous network nodes, which are distributed and connected in terms of a specific coupling structure [1,2,3,4]. CNs have attracted sustained interest from researchers in the field of systems and control due to their remarkable description/abstraction ability in numerous practical networks, including gene networks, power grids, transport networks, and artificial neural networks, and some representative results are provided in the references [5,6,7,8,9]. In the last decade, there has been a large body of research dedicated to investigating the issues in the dynamic analysis of CNs, including synchronization issues [10,11], stability analyses issues [12,13], state estimation (SE) issues [14,15], and others. It is important to highlight that studying CNs requires access to state information within the various nodes. However, measuring the network state directly from the sensor output measurements can pose challenges, especially in large-scale networks where information may be incomplete. Hence, the issues in SE for CNs, including variance-constrained SE, non-fragile SE, $H_{\infty }$ SE, minimum-variance SE, and recursive SE [16,17,18,19,20], have attracted a substantial amount of attention. For example, the variance-constrained SE problem has been investigated in [16] for a class of CNs involving quantized measurements and uncertain inner coupling. In [19], the randomly occurring sensor saturations were the focus, and a non-fragile state estimator of CNs was designed based on a dynamic event-triggering mechanism.

In practical networked control systems (NCSs), constraint phenomena inevitably occur due to the limited bandwidth, sensor failures, and complex operating environments, which may include system constraints (nonlinearity, time-delays, input saturation, and noise), measurement constraints (sensor saturation and sensor faults), and communication constraints (cyber attacks and channel fadings) [21,22,23,24,25,26,27,28]. The overall performance of CNs may be impacted more significantly by the constraint phenomena due to their intricate structure, which includes a topology of different network nodes and a coupling structure within a single node [29,30]. It is widely recognized that the outer coupling of a CN reflects the topology linkage among neighboring nodes, and the inner coupling depicts the intensity of the internal connections in an individual node. However, the coupling matrices are sometimes variable or unknown due to possible errors in modeling CNs, component aging, and changing working conditions [31]. Although the complexity of the coupling matrix makes it harder to analyze the dynamics of CNs, it is essential to take account of the impact of random couplings while analyzing the dynamic behavior of CNs. Research on constraint phenomena in CNs has received increasing interest [32,33,34]. For instance, the phenomena of uncertain inner coupling, sensor saturation, quantization, and missing measurements were contemplated regarding the SE problem for CNs in [34]. Due to the inadequate research results obtained, it is meaningful to conduct in-depth studies on the SE issue for CNs using random couplings, which is an objective of this paper.

For NCSs, communication transmission often takes up a large number of network resources, and the transmission data may be stolen by cyber hackers; thus, reducing the consumption of network resources in the communication process and ensuring the security of the data being communicated has become an urgent need. Compared to information transmission using analog signals among NCSs devices, communicating in a digital way has benefits including powerful resistance to noise disturbances, the high reliability of data transmission, and high confidentiality [35,36,37,38,39,40,41]. To date, various communication schemes have been adopted to decrease the consumption of communication resources, such as the communication protocol [42,43,44,45,46,47] and the event-triggering strategy [48,49,50]. Most existing data transmission strategies aim to alleviate the congestions and conflicts associated with transmission by reducing the communication traffic in the transmission channels or the frequency of data transmission.

In recent years, another communication mechanism has been widely studied, i.e., the encoding–decoding scheme [51,52,53,54], where the binary encoding scheme (BES), a traditional method, has been applied in the SE issue of NCSs [15,55,56]. The core idea of the BESs is to encode the data through a binary encoder as a binary bit string (BBS), which is to be transmitted via a binary symmetric channel (BSC) and then decoded by a binary decoder at the receiving end of the data. The use of BESs for transmission has allowed for data compression, reduced the communication bandwidth to an extent, and enhanced the security of data communication. Despite these advantages, distortion also occurs during data transmission using BESs. During the transmission of the BBS through a BSC, bit flipping inevitably occurs to a binary bit due to the presence of channel noise, e.g., a change from 0 to 1 or a change from 1 to 0. This phenomenon is also known as the bit error, which brings additional challenges to the analysis of CNs dynamics. If not dealt with properly when conducting the analysis, bit error may have a negative effect on both the correctness of data transmission and, inevitably, the estimator performance [15,56,57,58]; furthermore, investigations into the estimation of CNs using BESs have only recently become the subject of research. Hence, another objective of this paper was to study the SE issue for CNs using BESs while analyzing the impact of random bit errors on the estimator performance.

As is known, non-fragile SE issues with NCSs typically arise regarding the possible occurrence of fluctuations in gain in the actual execution course, which are caused by working environment effects or abrupt changes. In most of the literature on SE for CNs, the results were obtained by assuming that the constructed state estimators are accurate and realizable [59,60,61]. However, this assumption may be unrealistic for practical systems due to various factors interfering with the actual process, such as the aging of electronic components within the device or the inaccurate implementation of the digital system. That is, the implemented estimator gain parameters may deviate from the expected value to an unpredictable degree, which would sometimes cause severe estimation errors in the system. Therefore, the development of a non-fragile estimator that can resist these gain perturbations is necessary; this could involve including gain variations prior to estimation in the estimator model. At present, the non-fragile estimation issue is receiving increasing attention [19,62,63,64,65]. For instance, in [64], a $H_{\infty }$ non-fragile estimator was designed to deal with the fault estimation issue for a type of system with time-varying parameters over sensor networks. Nevertheless, non-fragile estimator designs for CNs have not been paid enough attention yet in which BESs, random couplings, and random multiple time delays are all taken into account simultaneously.

In response to these issues, our study aims to address the non-fragile SE problem for delayed CNs with randomly varying couplings under BESs. The crucial difficulties to be resolved through the developed non-fragile estimation strategy are as follows: (1) How can one characterize and describe the randomly varying couplings of CNs? (2) How can the influence from bit errors on SE performance be indicated? (3) How can a non-fragile estimator be devised that satisfies the exponential ultimate boundedness in mean squares? *The highlights of this paper are emphasized as follows: (1) the non-fragile SE issue is effectively tackled in this paper for nonlinear CNs, which encompass randomly occurring multiple delays and randomly varying couplings under the BESs; (2) a sufficient condition is brought up, which is used to design the non-fragile estimator, guarantee the exponential ultimate boundedness of the SE error dynamics in mean square, and derive the minimized ultimate bound of the estimation error; and (3) the estimator gain parameters are acquired via tackling an optimization issue constrained by some linear matrix inequalities.*

**Notation**: The symbol appearing in this paper is fairly normalised. $R^{n}$ is the *n*-dimensional Euclidean space. ∥·∥ indicates the standard norm. For a square matrix *A*, $A^{-1}$, tr{A}, $\varphi_{\min}\left(A\right)$ ($\varphi_{\max}\left(A\right)$), and A>0 (A≥0) reveal the inverse of *A*, the trace of *A*, the minimum (maximum) eigenvalue of *A*, and *A* is positive-definite (positive semi-definite), respectively. $B^{T}$ means the transpose of matrix *B*. The symbol ⊗ expresses the Kronecker product. $I_{\left(n\right)}$ ($0_{\left(r\times o\right)}$) represents the identity (zero) matrix with the dimension n×n (r×o). For a random variable/vector *w*, $V\left\{w\right\}$ and $E\left\{w\right\}$ symbolise its variance and expectation, respectively. diag{…} describes a diagonal matrix.

## 2. Problem Formulation and Preliminaries

### 2.1. The Mathematical Description of Network Nodes

A class of discrete-time delayed CNs with *U* nodes were used, as follows:

(1) $$\begin{array}{cc} x_{i}\left(s+1\right)= & A_{i}x_{i}\left(s\right)+B_{bi}\sum_{l=1}^{m}{϶}_{il}\left(s\right)x_{i}\left(s-b_{l}\left(s\right)\right)+E_{i}g\left(x_{i}\left(s\right)\right)\\ \; & +\sum_{\zeta =1}^{p}\sum_{j=1}^{U}\delta \left(\varkappa \left(s\right),\zeta \right)a_{ij}^{\left(\zeta \right)}\Gamma^{\left(\zeta \right)}x_{j}\left(s\right)+F_{i}w_{i}\left(s\right)\;\left(i=1,2,\ldots ,U\right) \end{array}$$

where $x_{i}\left(s\right)\in R^{l_{x}}$ denotes the state vector of node *i*; $w_{i}\left(s\right)\in R^{l_{w}}$ is the process noise depicted by a bounded stochastic noise sequence fulfilling $E\left\{w_{i}\left(s\right)\right\}=0$ and $V\left\{w_{i}\left(s\right)\right\}=w_{0}^{2}I_{\left(l_{w}\right)}$ with a scalar $w_{0}>0$; $A_{i}$, $B_{bi}$, $E_{i}$ and $F_{i}$ are the given system parameters matrices with suitable dimensions; and $\Lambda^{\left(\zeta \right)}={\left[a_{ij}^{\left(\zeta \right)}\right]}_{U\times U}\;\left(\zeta =1,2,\ldots ,p\right)$ is the outer coupling configuration matrix of CN (1) with $a_{ij}^{\left(\zeta \right)}\geq 0$ (i≠j) but not all zero. Generally, $\Lambda^{\left(\zeta \right)}$ is symmetric (i.e., $\Lambda^{\left(\zeta \right)}=\Lambda^{(\zeta )T}$ ) and satisfies

$$\begin{array}{c} \sum_{j=1}^{U}a_{ij}^{\left(\zeta \right)}=\sum_{j=1}^{U}a_{ji}^{\left(\zeta \right)}=0\;\left(i=1,2,\ldots ,U,\;\zeta =1,2,\ldots ,p\right). \end{array}$$

$\Gamma^{\left(\zeta \right)}=\mathrm{diag}\left\{\alpha_{1}^{\left(\zeta \right)},\alpha_{2}^{\left(\zeta \right)},\ldots ,\alpha_{l_{x}}^{\left(\zeta \right)}\right\}\geq 0$ is the inner coupling matrix linking the *ℏ*th $\left(\hbar =1,2,\ldots ,l_{x}\right)$ state variable if $\alpha_{\hbar }\neq 0$; δ(ϰ(s),ζ) is the Kronecker delta function, as follows:

$$\begin{array}{c} \delta \left(ı,℘\right)=\left\{\begin{array}{cc} 1, & \mathrm{if}\;ı=℘\\ 0, & \mathrm{if}\;ı\neq ℘. \end{array}\right. \end{array}$$

ϰ(s) is a stochastic variable that represents randomly varying couplings of CN (1), which conforms to a discrete-time homogeneous Markov chain with values in a finite state space, as follows:

$$\begin{array}{c} ℑ=\{1,2,\ldots ,p\} \end{array}$$

and $\Pi ={\left[\pi_{ab}\right]}_{p\times p}$ symbolises a transition probability matrix with elements denoted by

(2) $$\begin{array}{c} \pi_{ab}=\mathrm{Prob}\left\{\varkappa \left(s+1\right)=b|\varkappa \left(s\right)=a\right\}. \end{array}$$

**Assumption** **1** ([5]). *$g\left(\cdot \right)\in R^{l_{x}}$ is a nonlinear vector-valued function with g(0)=0, which meets*

(3) $$\begin{array}{cc} \; & {\left[g\left(z_{1}\right)-g\left(z_{2}\right)-\Xi_{1}\left(z_{1}-z_{2}\right)\right]}^{T}\left[g\left(z_{1}\right)-g\left(z_{2}\right)-\Xi_{2}\left(z_{1}-z_{2}\right)\right]\leq 0,\;\forall z_{1},z_{2}\in R^{l_{x}}. \end{array}$$

In the term $\sum_{l=1}^{m}{϶}_{il}\left(s\right)x_{i}\left(s-b_{l}\left(s\right)\right),\;b_{l}\left(s\right)\;\left(l=1,2,\ldots ,m\right)$ characterizes the stochastic and time-varying essence of communication delays that meet the condition $0<\underline{b}\;\leq \;b_{l}\left(s\right)\;\leq \;\bar{b}$, where $\underline{b}$ and $\bar{b}$ are known and stand for the lower and upper bounds of delays. ${϶}_{il}\left(s\right)$ is a random variable represented by a Bernoulli distribution, as follows:

$$\begin{array}{c} \mathrm{Prob}\left\{{϶}_{il}\left(s\right)=1\right\}={\bar{϶}}_{il},\;\mathrm{Prob}\left\{{϶}_{il}\left(s\right)=0\right\}=1-{\bar{϶}}_{il}. \end{array}$$

Denoting ${\tilde{϶}}_{il}\left(s\right)≜{϶}_{il}\left(s\right)-{\bar{϶}}_{il}$, we can obtain the following statistical properties:

$$\begin{array}{c} E\left\{{\left({϶}_{il}\left(s\right)-{\bar{϶}}_{il}\right)}^{2}\right\}=E\left\{{\left({\tilde{϶}}_{il}\left(s\right)\right)}^{2}\right\}={\bar{϶}}_{il}\left(1-{\bar{϶}}_{il}\right). \end{array}$$

Therefore, the dynamics of CN (1) can be rewritten as follows:

(4) $$\begin{array}{cc} x_{i}\left(s+1\right)= & A_{i}x_{i}\left(s\right)+\sum_{\zeta =1}^{p}\sum_{j=1}^{U}\delta \left(\varkappa \left(s\right),\zeta \right)a_{ij}^{\left(\zeta \right)}\Gamma^{\left(\zeta \right)}x_{j}\left(s\right)+B_{bi}\sum_{l=1}^{m}{\tilde{϶}}_{il}\left(s\right)x_{i}\left(s-b_{l}\left(s\right)\right)\\ \; & +B_{bi}\sum_{l=1}^{m}{\bar{϶}}_{il}x_{i}\left(s-b_{l}\left(s\right)\right)+E_{i}g\left(x_{i}\left(s\right)\right)+F_{i}w_{i}\left(s\right). \end{array}$$

The measurement signal $y_{i}\left(s\right)$ from the *i*th node is given as follows:

(5) $$\begin{array}{c} y_{i}\left(s\right)=C_{i}x_{i}\left(s\right)+D_{i}v_{i}\left(s\right) \end{array}$$

where $y_{i}\left(s\right)\in R^{l_{y}}$ is the measurement output of the *i*th (i=1,2,…,U) node, $C_{i}$ and $D_{i}$ represent known matrices with rational dimensions, and $v_{i}\left(s\right)\in R^{l_{v}}$ is the measurement noise, which is a bounded stochastic noise sequence satisfying $E\left\{v_{i}\left(s\right)\right\}=0$ and $V\left\{v_{i}\left(s\right)\right\}=v_{0}^{2}I_{\left(l_{v}\right)}$, with $v_{0}$ being a positive scalar.

### 2.2. The Adoption of BESs

Considering its anti-interference features and the security of its digital communication, we chose to use the BESs during the network transmission of measurement signals $y_{i}\left(s\right)$ (i=1,2,…,U) for CN (1). Suppose that the amplitude scope of a scalar signal $y_{i\iota }\left(s\right)$ ($\iota =1,2,\ldots ,l_{y}$) at instant *s* is [−τ,τ], where τ∈R>0 is a given scalar. A binary encoder was adopted to encode the measurement signal into a BBS with length *M*. Specifically, a uniform quantizer was utilized to quantize the output signal $y_{i\iota }\left(s\right)$ as ${\vec{y}}_{i\iota }\left(s\right)$, which corresponds to a BBS [66]. The whole measurement signal scope was divided into $2^{M}$ portions with a uniform interval size ρ, defined as follows:

(6) $$\begin{array}{c} \rho =\frac{2\tau }{2^{M}}. \end{array}$$

**Figure 1:**
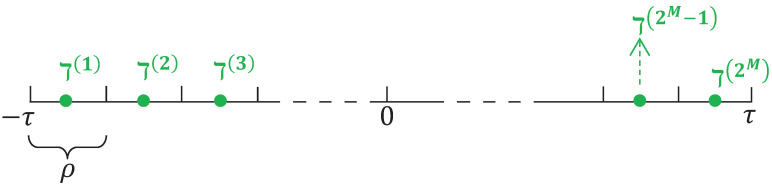
The set of quantization values (O).

Therefore, we obtained $2^{M}$ points (midpoint of every interval shown in Figure 1) defined by

$$O≜\{{ℸ}^{\left(1\right)},{ℸ}^{\left(2\right)},\ldots ,{ℸ}^{\left(2^{M}\right)}\},$$

where these points are spaced uniformly and each point is expressed using the following form:

$$\begin{array}{c} {ℸ}^{\left(\epsilon \right)}≜-\tau +\left(\epsilon -\frac{1}{2}\right)\rho ,\;\epsilon =1,2,\ldots ,2^{M}. \end{array}$$

The quantized signals were encoded by the encoder as BBSs and transferred through the BSC, and may flip randomly; they were described by a Bernoulli-distributed random variable with a probability *q* owing to the presence of channel noise [66]. The decoder recovered the received BBSs at the estimator side.

**Lemma** **1** ([66]). *Define $\xi_{i\iota }\left(s\right)≜{\vec{y}}_{i\iota }\left(s\right)-y_{i\iota }\left(s\right)$ as the quantization error. $\xi_{i\iota }\left(s\right)$ is an uniformly distributed stochastic variable in [−0.5ρ,0.5ρ] satisfying*

$$\begin{array}{c} E\left\{\xi_{i\iota }\left(s\right)\right\}=0,\;V\left\{\xi_{i\iota }\left(s\right)\right\}=\frac{\rho^{2}}{12}≜\xi_{0}^{2}<\frac{\rho^{2}}{4}=\frac{\tau^{2}}{2^{2M}}. \end{array}$$

**Lemma** **2** ([67]). *Define ${\overset{˘}{y}}_{i\iota }\left(s\right)$ as the decoder output signal and $h_{i\iota }\left(s\right)≜\frac{1}{1-2q}{\overset{˘}{y}}_{i\iota }\left(s\right)-{\vec{y}}_{i\iota }\left(s\right)$ as the equivalent noise reflecting impact from bit errors. $h_{i\iota }\left(s\right)$ has the following statistic property:*

$$\begin{array}{c} E\left\{h_{i\iota }\left(s\right)\right\}=0,\;V\left\{h_{i\iota }\left(s\right)\right\}≜h_{0}^{2}=\frac{4q\left(1-q\right)\left(2^{2M}-1\right)\tau^{2}}{3{\left(1-2q\right)}^{2}{\left(2^{M}-1\right)}^{2}}. \end{array}$$

In view of the aforementioned analysis, the signal used at the estimator is shown as follows:

(7) $$\begin{array}{c} {\tilde{y}}_{i}\left(s\right)=y_{i}\left(s\right)+\xi_{i}\left(s\right)+h_{i}\left(s\right). \end{array}$$

**Remark** **1.** *The uniform quantization approach, unlike probabilistic quantization (a type of random quantization), is a deterministic one that divides the scope of the signal into equally spaced quantization intervals. Typically, the midpoint of each interval is chosen as the quantized discrete value. When the length of the BBS in both [66,67] is B, the quantization error of uniform quantization in [66] seems to be reduced compared to that of probabilistic quantization in [67], and the variance bound of the quantization error in [67] is bigger (i.e., $\frac{\tau^{2}}{{\left(2^{M}-1\right)}^{2}}$) in comparison with that in [66].*

**Remark** **2.** *In (7), the description of the signal that is actually collected by the estimator is obtained via the measurement signal $y_{i}\left(s\right)$, the quantization error $\xi_{i}\left(s\right)$, and the equivalent noise-reflecting bit errors $h_{i}\left(s\right)$; this expression is beneficial to the establishment of the estimator model.*

**Remark** **3.** *The frequency of the occurrence of bit errors is measured using the bit error rate (BER), which is the percentage corresponding to the ratio of the number of flipping bits to the overall number of transferred binary bits. Here, we equate the probability of crossover to the BER. Note that bit errors may cause measurement deviations, reduce the realness and correctness of communication, and ultimately lead to a decrease in estimator performance.*

### 2.3. The Model Establishment of Non-Fragile State Estimator

Based on the received signal ${\tilde{y}}_{i}\left(s\right)$ (i=1,2,…,U) (7), a non-fragile state estimator was modelled for CN (1) as follows:

(8) $$\begin{array}{cc} {\hat{x}}_{i}\left(s+1\right) & =A_{i}{\hat{x}}_{i}\left(s\right)+\sum_{\zeta =1}^{p}\sum_{j=1}^{U}\delta \left(\varkappa \left(s\right),\zeta \right)a_{ij}^{\left(\zeta \right)}\Gamma^{\left(\zeta \right)}{\hat{x}}_{j}\left(s\right)+B_{bi}\sum_{l=1}^{m}{\bar{϶}}_{il}{\hat{x}}_{i}\left(s-b_{l}\left(s\right)\right)\\ \; & \;\;\;+E_{i}g\left({\hat{x}}_{i}\left(s\right)\right)+S_{i}\left(\varkappa \left(s\right)\right)\left(I_{\left(l_{y}\right)}+▵\left(s\right)\right)\left({\tilde{y}}_{i}\left(s\right)-C_{i}{\hat{x}}_{i}\left(s\right)\right) \end{array}$$

where ${\hat{x}}_{i}\left(s\right)\in R^{l_{x}}$ denotes the estimate of $x_{i}\left(s\right)$, and $S_{i}\left(\varkappa \left(s\right)\right)$ (i=1,2,…,U) describes the unknown estimator gain, which is determined later.

In (8), the gain variation is considered and represented as ▵(s), which has the following specific form:

(9) $$\begin{array}{c} ▵(s)=mH(s)n \end{array}$$

where m and n are given matrix parameters with rational dimensions, and H(s) is an uncertain matrix that satisfies $H^{T}\left(s\right)H\left(s\right)\leq I_{\left(l_{y}\right)}$.

Setting $e_{i}\left(s\right)≜x_{i}\left(s\right)-{\hat{x}}_{i}\left(s\right)$ and $\bar{g}\left(e_{i}\left(s\right)\right)≜g\left(x_{i}\left(s\right)\right)-g\left({\hat{x}}_{i}\left(s\right)\right)$, the acquired estimation error is as follows:

(10) $$\begin{array}{cc} e_{i}\left(s+1\right)= & (A_{i}-S_{i}\left(\varkappa \left(s\right)\right)\left(I_{\left(l_{y}\right)}+▵\left(s\right)C_{i}\right)e_{i}\left(s\right)+\sum_{\zeta =1}^{p}\sum_{j=1}^{U}\delta \left(\varkappa \left(s\right),\zeta \right)a_{ij}^{\left(\zeta \right)}\Gamma^{\left(\zeta \right)}e_{j}\left(s\right)\\ \; & +B_{bi}\sum_{l=1}^{m}{\tilde{϶}}_{il}\left(s\right)x_{i}\left(s-b_{l}\left(s\right)\right)+B_{bi}\sum_{l=1}^{m}{\bar{϶}}_{il}e_{i}\left(s-b_{l}\left(s\right)\right)+E_{i}\bar{g}\left(e_{i}\left(s\right)\right)\\ \; & +F_{i}w_{i}\left(s\right)-S_{i}\left(\varkappa \left(s\right)\right)\left(I_{\left(l_{y}\right)}+▵\left(s\right)\right)D_{i}v_{i}\left(s\right)-S_{i}\left(\varkappa \left(s\right)\right)\left(I_{\left(l_{y}\right)}+▵\left(s\right)\right)\xi_{i}\left(s\right)\\ \; & -S_{i}\left(\varkappa \left(s\right)\right)\left(I_{\left(l_{y}\right)}+▵\left(s\right)\right)h_{i}\left(s\right). \end{array}$$

To simplify the expression, we present the following notations:

$$\begin{array}{cc} O & ≜\mathrm{diag}\left\{O_{1},O_{2},\ldots ,O_{U}\right\}\;\left(O=A,B_{b},C,E,D,F\right),\\ S(\varkappa (s)) & ≜\mathrm{diag}\{S_{1}\left(\varkappa \left(s\right)\right),S_{2}\left(\varkappa \left(s\right)\right),\ldots ,S_{U}\left(\varkappa \left(s\right)\right)\},\\ n(s) & ≜{\left[n_{1}^{T}\left(s\right)\;n_{2}^{T}\left(s\right)\;\ldots \;n_{U}^{T}\left(s\right)\right]}^{T}\;\left(n=x,e,w,v,\xi ,h\right),\\ g(x(s)) & ≜{\left[\begin{array}{cccc} g^{T}\left(x_{1}\left(s\right)\right) & g^{T}\left(x_{2}\left(s\right)\right) & \cdots & g^{T}\left(x_{U}\left(s\right)\right) \end{array}\right]}^{T},\\ \tilde{g}\left(e\left(s\right)\right) & ≜{\left[\begin{array}{cccc} {\bar{g}}^{T}\left(e_{1}\left(s\right)\right) & {\bar{g}}^{T}\left(e_{2}\left(s\right)\right) & \cdots & {\bar{g}}^{T}\left(e_{U}\left(s\right)\right) \end{array}\right]}^{T},\\ {\tilde{\Theta }}_{l}\left(s\right) & ≜\mathrm{diag}\{{\tilde{϶}}_{1l}\left(s\right)I_{\left(l_{x}\right)},{\tilde{϶}}_{2l}\left(s\right)I_{\left(l_{x}\right)},\ldots ,{\tilde{϶}}_{Ul}\left(s\right)I_{\left(l_{x}\right)}\},\\ {\bar{\Theta }}_{l} & ≜\mathrm{diag}\{{\bar{϶}}_{1l}I_{\left(l_{x}\right)},{\bar{϶}}_{2l}I_{\left(l_{x}\right)},\ldots ,{\bar{϶}}_{Ul}I_{\left(l_{x}\right)}\},\\ x(s-b_{l}\left(s\right)) & ≜{\left[\begin{array}{cccc} x_{1}^{T}\left(s-b_{l}\left(s\right)\right) & x_{2}^{T}\left(s-b_{l}\left(s\right)\right) & \cdots & x_{U}^{T}\left(s-b_{l}\left(s\right)\right) \end{array}\right]}^{T},\\ e(s-b_{l}\left(s\right)) & ≜{\left[\begin{array}{cccc} e_{1}^{T}\left(s-b_{l}\left(s\right)\right) & e_{2}^{T}\left(s-b_{l}\left(s\right)\right) & \cdots & e_{U}^{T}\left(s-b_{l}\left(s\right)\right) \end{array}\right]}^{T}. \end{array}$$

Compact forms of (4) and (10) are attained as follows:

(11) $$\begin{array}{cc} x(s+1) & =Ax\left(s\right)+\sum_{\zeta =1}^{p}\delta \left(\varkappa \left(s\right),\zeta \right)\Lambda^{\left(\zeta \right)}⊗\Gamma^{\left(\zeta \right)}x\left(s\right)+\sum_{l=1}^{m}{\tilde{\Theta }}_{l}\left(s\right)B_{b}x\left(s-b_{l}\left(s\right)\right)\\ \; & \;\;\;+\sum_{l=1}^{m}{\bar{\Theta }}_{l}B_{b}x\left(s-b_{l}\left(s\right)\right)+Eg\left(x\left(s\right)\right)+Fw\left(s\right) \end{array}$$

and

(12) $$\begin{array}{cc} e(s+1) & =\left(\sum_{\zeta =1}^{p}\delta \left(\varkappa \left(s\right),\zeta \right)\Lambda^{\left(\zeta \right)}⊗\Gamma^{\left(\zeta \right)}+A-S\left(\varkappa \left(s\right)\right)I_{▵}\left(s\right)C\right)e\left(s\right)\\ \; & \;\;\;+\sum_{l=1}^{m}{\tilde{\Theta }}_{l}\left(s\right)B_{b}x\left(s-b_{l}\left(s\right)\right)+\sum_{l=1}^{m}{\bar{\Theta }}_{l}B_{b}e\left(s-b_{l}\left(s\right)\right)\\ \; & \;\;\;+E\tilde{g}\left(e\left(s\right)\right)+Fw\left(s\right)-S\left(\varkappa \left(s\right)\right)I_{▵}\left(s\right)Dv\left(s\right)\\ \; & \;\;\;-S\left(\varkappa \left(s\right)\right)I_{▵}\left(s\right)\xi \left(s\right)-S\left(\varkappa \left(s\right)\right)I_{▵}\left(s\right)h\left(s\right) \end{array}$$

where

$$\begin{array}{c} I_{▵}\left(s\right)≜I_{\left(Ul_{y}\right)}+I_{\left(U\right)}⊗▵\left(s\right). \end{array}$$

Let $\eta \left(s\right)≜{\left[\begin{array}{cc} x^{T}\left(s\right) & e^{T}\left(s\right) \end{array}\right]}^{T}$ and $\overset{ˇ}{g}\left(\eta \left(s\right)\right)≜{\left[\begin{array}{cc} g^{T}\left(x\left(s\right)\right) & {\tilde{g}}^{T}\left(e\left(s\right)\right) \end{array}\right]}^{T}$. The following augmented SE error dynamics were derived with regard to (11)–(12):

(13) $$\begin{array}{cc} \eta (s+1) & =A\left(\varkappa \left(s\right)\right)\eta \left(s\right)+\sum_{l=1}^{m}{\overset{˘}{\Theta }}_{l}\left(s\right)B_{b1}\eta \left(s-b_{l}\left(s\right)\right)+\sum_{l=1}^{m}{\overset{ˇ}{\Theta }}_{l}B_{b2}\eta \left(s-b_{l}\left(s\right)\right)\\ \; & \;\;\;+E\overset{ˇ}{g}\left(\eta \left(s\right)\right)+Fw\left(s\right)-S\left(\varkappa \left(s\right)\right)I_{▵}\left(s\right)\left(Dv\left(s\right)+\xi \left(s\right)+h\left(s\right)\right) \end{array}$$

where

$$\begin{array}{cc} A(\varkappa (s))≜ & \mathrm{diag}\{A+\sum_{\zeta =1}^{p}\delta \left(\varkappa \left(s\right),\zeta \right)\Lambda^{\left(\zeta \right)}⊗\Gamma^{\left(\zeta \right)},\sum_{\zeta =1}^{p}\delta \left(\varkappa \left(s\right),\zeta \right)\Lambda^{\left(\zeta \right)}⊗\Gamma^{\left(\zeta \right)}+A-S\left(\varkappa \left(s\right)\right)\\ \; & \times I_{▵}\left(s\right)C\},\\ B_{b1}≜ & \left[\begin{array}{cc} B_{b} & 0_{\left(\left(Ul_{x}\right)\times \left(Ul_{x}\right)\right)}\\ B_{b} & 0_{\left(\left(Ul_{x}\right)\times \left(Ul_{x}\right)\right)} \end{array}\right],B_{b2}≜\mathrm{diag}\left\{B_{b},B_{b}\right\},S\left(\varkappa \left(s\right)\right)≜\left[\begin{array}{c} 0_{\left(\left(Ul_{x}\right)\times \left(Ul_{y}\right)\right)}\\ S(\varkappa (s)) \end{array}\right],\\ E≜ & \mathrm{diag}\left\{E,E\right\},\;{\overset{˘}{\Theta }}_{l}\left(s\right)≜\mathrm{diag}\left\{{\tilde{\Theta }}_{l}\left(s\right),{\tilde{\Theta }}_{l}\left(s\right)\right\},\;{\overset{ˇ}{\Theta }}_{l}≜\mathrm{diag}\left\{{\bar{\Theta }}_{l},{\bar{\Theta }}_{l}\right\},F≜{\left[\begin{array}{cc} F & F \end{array}\right]}^{T}. \end{array}$$

**Definition** **1** ([5]). *If there exist scalars ϕ>0, 0≤ψ<1 and m>0 which fulfill*

(14) $$\begin{array}{c} E\left\{{∥\eta (s)∥}^{2}\right\}\leq \phi \psi^{s}\underset{-\bar{b}\leq ȷ\leq 0}{\sup}{E\{∥\eta \left(ȷ\right)∥}^{2}\}+m, \end{array}$$

*then the state estimator (8) is called an exponentially ultimately bounded (EUB) estimator in the mean square for CN (1).*

**Figure 2:**
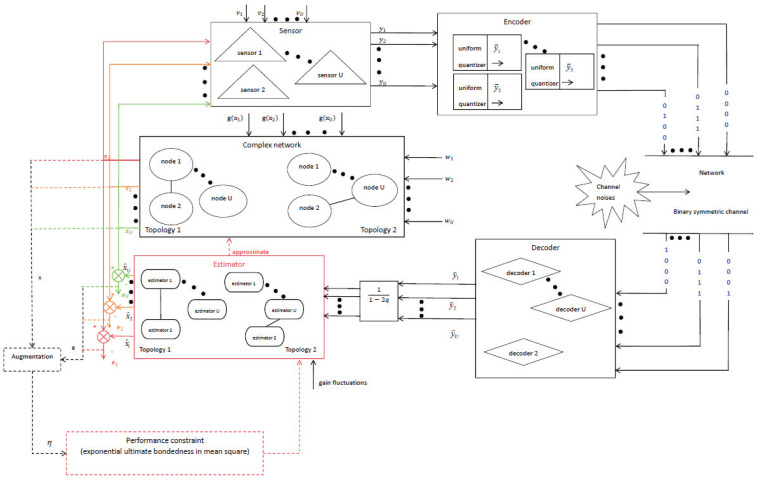
The diagram of state estimation (SE) for complex network (CN) (1).

The goal of this paper is to design an EUB non-fragile state estimator (8) in mean square for CN (1). The SE issue discussed in this paper is shown in Figure 2.

## 3. Main Results

A few Lemmas are illustrated as follows; these were used for the performance analysis and estimator design (8).

**Lemma** **3** ([68]). *Considering a matrix P>0, and vectors $L^{T}$ and $U^{T}$, the following inequality holds:*

$$\begin{array}{c} L^{T}PU+U^{T}PL\leq L^{T}PL+U^{T}PU \end{array}$$

*holds.*

**Lemma** **4** ([5]). *Constants 0≤f≤1 and γ are provided. If {J(s)|s=0,1,2,…} is a nonnegative sequence with*

$$\begin{array}{c} J(s+1)\leq fJ(s)+\gamma , \end{array}$$

*then,*

$$\begin{array}{c} J\left(s\right)\leq \left\{\begin{array}{cc} J(0)+s\gamma & \mathrm{for}\;f=1,\\ f^{s}J\left(0\right)+\frac{\gamma (1-f^{s})}{1-f} & \mathrm{for}\;f\neq 1. \end{array}\right. \end{array}$$

**Lemma** **5** ([64]). *Let matrices $X=X^{T}$, E and J be of suitable sizes, and F meets $FF^{T}\leq I$; then, we can obtain that $X+JFE+E^{T}F^{T}J^{T}\leq 0$ is met if and only if there is a scalar ε>0 satisfying $X+\varepsilon^{-1}JJ^{T}+\varepsilon E^{T}E<0$ or*

$$\begin{array}{c} \left[\begin{array}{ccc} X & * & *\\ J^{T} & -\varepsilon I & *\\ \varepsilon E & 0 & -\varepsilon I \end{array}\right]<0. \end{array}$$

Now, we will analyze the estimator performance for CN (1).

**Theorem** **1.** *Consider that the scalar 0<κ<1 and the gain parameter $S_{i}\left(\varkappa \left(s\right)\right)$ (i=1,2,…,U, ϰ(s)∈ℑ) are known. The estimator (8) becomes an EUB of CN (1) in mean square if matrices $P_{a}≜\mathrm{diag}\left\{P_{1a},P_{2a}\right\}>0$ $(P_{1a}≜\mathrm{diag}\left\{P_{11a},P_{12a},\ldots ,P_{1Ua}\right\}$, $P_{2a}≜\mathrm{diag}\left\{P_{21a},P_{22a},\ldots ,P_{2Ua}\right\})$, $Q_{v}≜\mathrm{diag}\left\{Q_{1v},Q_{2v}\right\}>0$ $(Q_{1v}≜\mathrm{diag}\{Q_{11v},Q_{12v},$$\ldots ,Q_{1Uv}\}$, $Q_{2v}≜\mathrm{diag}\{Q_{21v},$$Q_{22v},\ldots ,Q_{2Uv}\})$ (v=1,2,…,m), and scalars $\lambda_{a}>0$ satisfy the following inequality:*

(15) $$\begin{array}{c} \Psi_{a}=\left[\begin{array}{ccc} \Psi_{11a} & \Psi_{12a} & \Psi_{13a}\\ {}* & \Psi_{22a} & \Psi_{23a}\\ {}* & * & \Psi_{33a} \end{array}\right]<0 \end{array}$$

*where*

$$\begin{array}{cc} \Psi_{11a}≜ & A^{T}{\bar{P}}_{a}A-\lambda_{a}{\tilde{\Xi }}_{1}+\left(1+\bar{b}-\underline{b}\right)\sum_{v=1}^{m}Q_{v}-P_{a},\;\Psi_{12a}≜A^{T}{\bar{P}}_{a}\left(\overset{ˇ}{\Theta }⊗B_{b2}\right),\\ {\bar{P}}_{a}≜ & \sum_{b=1}^{p}\pi_{ab}P_{b},\;\Psi_{13a}≜A^{T}{\bar{P}}_{a}E+\lambda_{a}{\tilde{\Xi }}_{2}^{T},\;{\tilde{\Xi }}_{1}≜I_{\left(2U\right)}⊗{\bar{\Xi }}_{1},\;{\tilde{\Xi }}_{2}≜I_{\left(2U\right)}⊗{\bar{\Xi }}_{2},\\ \Psi_{22a}≜ & {\left(I_{\left(m\right)}⊗B_{b1}\right)}^{T}{\hat{\Theta }}^{T}\left(I_{\left(m\right)}⊗{\bar{P}}_{a}\right)\hat{\Theta }\left(I_{\left(m\right)}⊗B_{b1}\right)+{\left(\overset{ˇ}{\Theta }⊗B_{b2}\right)}^{T}\bar{P_{a}}\left(\overset{ˇ}{\Theta }⊗B_{b2}\right)-{\left(1-\kappa \right)}^{\bar{b}}\bar{Q},\\ \Psi_{23a}≜ & {\left(\overset{ˇ}{\Theta }⊗B_{b2}\right)}^{T}{\bar{P}}_{a}E,\;\Psi_{33a}≜E^{T}{\bar{P}}_{a}E-\lambda_{a}I_{\left(2Ul_{x}\right)},\;\overset{ˇ}{\Theta }≜\left[\begin{array}{cccc} {\overset{ˇ}{\Theta }}_{1} & {\overset{ˇ}{\Theta }}_{2}\; & \cdots & {\overset{ˇ}{\Theta }}_{m} \end{array}\right],\\ {\bar{\Xi }}_{1}≜ & \frac{\Xi_{1}^{T}\Xi_{2}+\Xi_{2}^{T}\Xi_{1}}{2},\;{\bar{\Xi }}_{2}≜\frac{\Xi_{1}+\Xi_{2}}{2},\;\bar{Q}≜\mathrm{diag}\left\{Q_{1},Q_{2},\ldots ,Q_{m}\right\},\\ {\hat{\Theta }}_{l}≜ & \mathrm{diag}\left\{\sqrt{{\bar{϶}}_{1l}\left(1-{\bar{϶}}_{1l}\right)}I_{\left(l_{x}\right)},\sqrt{{\bar{϶}}_{2l}\left(1-{\bar{϶}}_{2l}\right)}I_{\left(l_{x}\right)},\ldots ,\sqrt{{\bar{϶}}_{Ul}\left(1-{\bar{϶}}_{Ul}\right)}I_{\left(l_{x}\right)}\right\},\\ \hat{\Theta }≜ & \mathrm{diag}\{I_{\left(2\right)}⊗{\hat{\Theta }}_{1},I_{\left(2\right)}⊗{\hat{\Theta }}_{2},\ldots ,I_{\left(2\right)}⊗{\hat{\Theta }}_{m}\}. \end{array}$$

**Proof** **of Theorem 1.** The Lyapunov functional is defined as follows:

(16) $$\begin{array}{c} V\left(s,\eta (s),\varkappa (s)\right)≜\sum_{r=1}^{3}V_{r}\left(s,\eta (s),\varkappa (s)\right) \end{array}$$

where

$$\begin{array}{cc} \; & V_{1}\left(s,\eta \left(s\right),\varkappa \left(s\right)\right)≜\eta^{T}\left(s\right)P\left(\varkappa \left(s\right)\right)\eta \left(s\right),\\ \; & V_{2}\left(s,\eta \left(s\right)\right)≜\sum_{v=1}^{m}\sum_{u=s-b_{v}\left(s\right)}^{s-1}{\left(1-\kappa \right)}^{s-u-1}\eta^{T}\left(u\right)Q_{v}\eta \left(u\right),\\ \; & V_{3}\left(s,\eta \left(s\right)\right)≜\sum_{v=1}^{m}\sum_{n=s-\bar{b}+1}^{s-\underline{b}}\sum_{u=n}^{s-1}{\left(1-\kappa \right)}^{s-u-1}\eta^{T}\left(u\right)Q_{v}\eta \left(u\right). \end{array}$$

Letting $P_{a}≜P\left(\varkappa (s)=a\right)$ (a∈ℑ), and noticing that $E\{{\overset{˘}{\Theta }}_{l}\left(s\right)\}=0$, E{w(s)}=0, E{v(s)}=0, E{ξ(s)}=0 and E{h(s)}=0, one can observe from (13) that

$$\begin{array}{cc} \; & E\left\{\Delta V_{1}\left(s,\eta \left(s\right),\varkappa \left(s\right)\right)\right\}=E\left\{V_{1}\left(s+1,\eta (s+1),\varkappa (s+1)\right)|\eta \left(s\right),\varkappa \left(s\right)=a\right\}-V_{1}\left(s,\eta \left(s\right),a\right)\\ = & E\{\eta^{T}\left(s\right)A^{T}{\bar{P}}_{a}A\eta \left(s\right)-\eta^{T}\left(s\right)P_{a}\eta \left(s\right)+2\eta^{T}\left(s\right)A^{T}{\bar{P}}_{a}E\overset{ˇ}{g}\left(\eta \left(s\right)\right)+2\eta^{T}\left(s\right)A^{T}{\bar{P}}_{a}\left(\overset{ˇ}{\Theta }⊗B_{b2}\right)\\ \; & \times \eta \left(s-b\left(s\right)\right)+{\overset{ˇ}{g}}^{T}\left(\eta \left(s\right)\right)E^{T}{\bar{P}}_{a}E\overset{ˇ}{g}\left(\eta \left(s\right)\right)+w^{T}\left(s\right)F^{T}{\bar{P}}_{a}Fw\left(s\right)+\eta^{T}\left(s-b\left(s\right)\right){\left(I_{\left(m\right)}⊗B_{b1}\right)}^{T}\\ \; & \times {\hat{\Theta }}^{T}\left(I_{\left(m\right)}⊗{\bar{P}}_{a}\right)\hat{\Theta }\left(I_{\left(m\right)}⊗B_{b1}\right)\eta \left(s-b\left(s\right)\right)+\eta^{T}\left(s-b\left(s\right)\right){\left(\overset{ˇ}{\Theta }⊗B_{b2}\right)}^{T}{\bar{P}}_{a}\left(\overset{ˇ}{\Theta }⊗B_{b2}\right)\\ \; & \times \eta \left(s-b\left(s\right)\right)+2\eta^{T}\left(s-b\left(s\right)\right){\left(\overset{ˇ}{\Theta }⊗B_{b2}\right)}^{T}{\bar{P}}_{a}E\overset{ˇ}{g}\left(\eta \left(s\right)\right)+v^{T}\left(s\right)D^{T}I_{▵}^{T}\left(s\right)S_{a}^{T}{\bar{P}}_{a}S_{a}I_{▵}\left(s\right)\\ \; & \times Dv\left(s\right)+\xi^{T}\left(s\right)I_{▵}^{T}\left(s\right)S_{a}^{T}{\bar{P}}_{a}S_{a}I_{▵}\left(s\right)\xi \left(s\right)+h^{T}\left(s\right)I_{▵}^{T}\left(s\right)S_{a}^{T}{\bar{P}}_{a}S_{a}I_{▵}\left(s\right)h\left(s\right)\\ \; & +2v^{T}\left(s\right)D^{T}I_{▵}^{T}\left(s\right)S_{a}^{T}{\bar{P}}_{a}S_{a}I_{▵}\left(s\right)\xi \left(s\right)+2v^{T}\left(s\right)D^{T}I_{▵}^{T}\left(s\right)S_{a}^{T}{\bar{P}}_{a}S_{a}I_{▵}\left(s\right)h\left(s\right)\\ \; & +2\xi^{T}\left(s\right)I_{▵}^{T}\left(s\right)S_{a}^{T}{\bar{P}}_{a}S_{a}I_{▵}\left(s\right)h\left(s\right)\}. \end{array}$$

Using Lemma 3, we can obtain the following inequalities:

$$\begin{array}{cc} \; & 2v^{T}\left(s\right)D^{T}I_{▵}^{T}\left(s\right)S_{a}^{T}{\bar{P}}_{a}S_{a}I_{▵}\left(s\right)\xi \left(s\right) \end{array}$$

(17) $$\begin{array}{cc} \leq & v^{T}\left(s\right)D^{T}I_{▵}^{T}\left(s\right)S_{a}^{T}{\bar{P}}_{a}S_{a}I_{▵}\left(s\right)Dv\left(s\right)+\xi^{T}\left(s\right)I_{▵}^{T}\left(s\right)S_{a}^{T}{\bar{P}}_{a}S_{a}I_{▵}\left(s\right)\xi \left(s\right),\\ \; & 2v^{T}\left(s\right)D^{T}I_{▵}^{T}\left(s\right)S_{a}^{T}{\bar{P}}_{a}S_{a}I_{▵}\left(s\right)h\left(s\right) \end{array}$$

(18) $$\begin{array}{cc} \leq & v^{T}\left(s\right)D^{T}I_{▵}^{T}\left(s\right)S_{a}^{T}{\bar{P}}_{a}S_{a}I_{▵}\left(s\right)Dv\left(s\right)+h^{T}\left(s\right)I_{▵}^{T}\left(s\right)S_{a}^{T}{\bar{P}}_{a}S_{a}I_{▵}\left(s\right)h\left(s\right),\\ \; & 2\xi^{T}\left(s\right)I_{▵}^{T}\left(s\right)S_{a}^{T}{\bar{P}}_{a}S_{a}I_{▵}\left(s\right)h\left(s\right) \end{array}$$

(19) $$\begin{array}{cc} \leq & \xi^{T}\left(s\right)I_{▵}^{T}\left(s\right)S_{a}^{T}{\bar{P}}_{a}S_{a}I_{▵}\left(s\right)\xi \left(s\right)+h^{T}\left(s\right)I_{▵}^{T}\left(s\right)S_{a}^{T}{\bar{P}}_{a}S_{a}I_{▵}\left(s\right)h\left(s\right) \end{array}$$

By utilizing the property Fℶ=ℶF (where F and *ℶ* are two diagonal matrices), and the characteristic of matrix trace (tr{WY}=tr{YW}, tr{c}=c (where c∈R is a constant) and tr{diag{W,Y}}=tr{W}+tr{Y}), and combining this with (17)–(19), we obtain the following:

(20) $$\begin{array}{cc} \; & E\left\{\Delta V_{1}\left(s,\eta \left(s\right),\varkappa \left(s\right)\right)\right\}\\ \leq & E\{\eta^{T}\left(s\right)A^{T}{\bar{P}}_{a}A\eta \left(s\right)-\eta^{T}\left(s\right)P_{a}\eta \left(s\right)+2\eta^{T}\left(s\right)A^{T}{\bar{P}}_{a}E\overset{ˇ}{g}\left(\eta \left(s\right)\right)+2\eta^{T}\left(s\right)A^{T}{\bar{P}}_{a}\left(\overset{ˇ}{\Theta }⊗B_{b2}\right)\\ \; & \times \eta \left(s-b\left(s\right)\right)+{\overset{ˇ}{g}}^{T}\left(\eta \left(s\right)\right)E^{T}{\bar{P}}_{a}E\overset{ˇ}{g}\left(\eta \left(s\right)\right)+\eta^{T}\left(s-b\left(s\right)\right){\left(I_{\left(m\right)}⊗B_{b1}\right)}^{T}{\hat{\Theta }}^{T}\left(I_{\left(m\right)}⊗{\bar{P}}_{a}\right)\hat{\Theta }\\ \; & \times \left(I_{\left(m\right)}⊗B_{b1}\right)\eta \left(s-b\left(s\right)\right)+\eta^{T}\left(s-b\left(s\right)\right){\left(\overset{ˇ}{\Theta }⊗B_{b2}\right)}^{T}{\bar{P}}_{a}\left(\overset{ˇ}{\Theta }⊗B_{b2}\right)\eta \left(s-b\left(s\right)\right)\\ \; & +2\eta^{T}\left(s-b\left(s\right)\right){\left(\overset{ˇ}{\Theta }⊗B_{b2}\right)}^{T}{\bar{P}}_{a}E\overset{ˇ}{g}\left(\eta \left(s\right)\right)\\ \; & +\mathrm{tr}\left\{F^{T}{\bar{P}}_{a}Fw\left(s\right)w^{T}\left(s\right)\right\}+\mathrm{tr}\left\{3D^{T}I_{▵}^{T}\left(s\right)S_{a}^{T}{\bar{P}}_{a}S_{a}I_{▵}\left(s\right)Dv\left(s\right)v^{T}\left(s\right)\right\}\\ \; & +\mathrm{tr}\{3I_{▵}^{T}\left(s\right)S_{a}^{T}{\bar{P}}_{a}S_{a}I_{▵}\left(s\right)\xi \left(s\right)\xi^{T}\left(s\right)+3I_{▵}^{T}\left(s\right)S_{a}^{T}{\bar{P}}_{a}S_{a}I_{▵}\left(s\right)h\left(s\right)h^{T}\left(s\right)\}\} \end{array}$$

where

$$\begin{array}{cc} \; & \;\;\;\eta \left(s-b\left(s\right)\right)≜{\left[\begin{array}{cccc} \eta^{T}\left(s-b_{1}\left(s\right)\right) & \eta^{T}\left(s-b_{2}\left(s\right)\right)\; & \cdots & \eta^{T}\left(s-b_{m}\left(s\right)\right) \end{array}\right]}^{T}, \end{array}$$

(21) $$\begin{array}{cc} \; & E\left\{\Delta V_{2}\left(s,\eta \left(s\right)\right)\right\}\\ = & E\left\{\sum_{v=1}^{m}\sum_{u=s-b_{v}\left(s+1\right)+1}^{s}{\left(1-\kappa \right)}^{s-u}\eta^{T}\left(u\right)Q_{v}\eta \left(u\right)-\sum_{v=1}^{m}\sum_{u=s-b_{v}\left(s\right)}^{s-1}{\left(1-\kappa \right)}^{s-u-1}\eta^{T}\left(u\right)Q_{v}\eta \left(u\right)\right\}\\ = & \sum_{v=1}^{m}\{\eta^{T}\left(s\right)Q_{v}\eta \left(s\right)+\sum_{u=s-b_{v}\left(s+1\right)+1}^{s-1}{\left(1-\kappa \right)}^{s-u}\eta^{T}\left(u\right)Q_{v}\eta \left(u\right)\\ \; & -\sum_{u=s-b_{v}\left(s\right)}^{s-1}{\left(1-\kappa \right)}^{s-u-1}\eta^{T}\left(u\right)Q_{v}\eta \left(u\right)\}\\ \leq & \sum_{v=1}^{m}\{\eta^{T}\left(s\right)Q_{v}\eta \left(s\right)+\sum_{u=s-\bar{b}+1}^{s-\underline{b}}{\left(1-\kappa \right)}^{s-u}\eta^{T}\left(u\right)Q_{v}\eta \left(u\right)+\sum_{u=s-b_{v}\left(s\right)+1}^{s-1}{\left(1-\kappa \right)}^{s-u}\eta^{T}\left(u\right)\\ \; & \times Q_{v}\eta \left(u\right)-\sum_{u=s-b_{v}\left(s\right)}^{s-1}{\left(1-\kappa \right)}^{s-u-1}\eta^{T}\left(u\right)Q_{v}\eta \left(u\right)\}\\ = & \sum_{v=1}^{m}\{\eta^{T}\left(s\right)Q_{v}\eta \left(s\right)+\sum_{u=s-\bar{b}+1}^{s-\underline{b}}{\left(1-\kappa \right)}^{s-u}\eta^{T}\left(u\right)Q_{v}\eta \left(u\right)+\sum_{u=s-b_{v}\left(s\right)}^{s-1}{\left(1-\kappa \right)}^{s-u}\eta^{T}\left(u\right)Q_{v}\\ \; & \times \eta \left(u\right)-{\left(1-\kappa \right)}^{b_{v}\left(s\right)}\eta^{T}\left(s-b_{v}\left(s\right)\right)Q_{v}\eta \left(s-b_{v}\left(s\right)\right)-\sum_{u=s-b_{v}\left(s\right)}^{s-1}{\left(1-\kappa \right)}^{s-u-1}\eta^{T}\left(u\right)Q_{v}\eta \left(u\right)\}\\ \leq & \sum_{v=1}^{m}\{\eta^{T}\left(s\right)Q_{v}\eta \left(s\right)+\sum_{u=s-\bar{b}+1}^{s-\underline{b}}{\left(1-\kappa \right)}^{s-u}\eta^{T}\left(u\right)Q_{v}\eta \left(u\right)-{\left(1-\kappa \right)}^{\bar{b}}\eta^{T}\left(s-b_{v}\left(s\right)\right)Q_{v}\\ \; & \times \eta \left(s-b_{v}\left(s\right)\right)-\kappa \sum_{u=s-b_{v}\left(s\right)}^{s-1}{\left(1-\kappa \right)}^{s-u-1}\eta^{T}\left(u\right)Q_{v}\eta \left(u\right)\}\\ = & \sum_{v=1}^{m}\{\eta^{T}\left(s\right)Q_{v}\eta \left(s\right)+\sum_{u=s-\bar{b}+1}^{s-\underline{b}}{\left(1-\kappa \right)}^{s-u}\eta^{T}\left(u\right)Q_{v}\eta \left(u\right)-{\left(1-\kappa \right)}^{\bar{b}}\eta^{T}\left(s-b_{v}\left(s\right)\right)Q_{v}\\ \; & \times \eta \left(s-b_{v}\left(s\right)\right)\}-\kappa V_{2}\left(s,\eta \left(s\right)\right) \end{array}$$

and

(22) $$\begin{array}{cc} \; & E\left\{\Delta V_{3}\left(s,\eta \left(s\right)\right)\right\}\\ = & E\{\sum_{v=1}^{m}\sum_{n=s-\bar{b}+2}^{s+1-\underline{b}}\sum_{u=n}^{s}{\left(1-\kappa \right)}^{s-u}\eta^{T}\left(u\right)Q_{v}\eta \left(u\right)-\sum_{v=1}^{m}\sum_{n=s-\bar{b}+1}^{s-\underline{b}}\sum_{u=n}^{s-1}{\left(1-\kappa \right)}^{s-u-1}\\ \; & \times \eta^{T}\left(u\right)Q_{v}\eta \left(u\right)\}\\ = & \sum_{v=1}^{m}\{\left(\bar{b}-\underline{b}\right)\eta^{T}\left(s\right)Q_{v}\eta \left(s\right)+\sum_{n=s-\bar{b}+2}^{s+1-\underline{b}}\sum_{u=n}^{s-1}{\left(1-\kappa \right)}^{s-u}\eta^{T}\left(u\right)Q_{v}\eta \left(u\right)\\ \; & -\sum_{n=s-\bar{b}+1}^{s-\underline{b}}\sum_{u=n}^{s-1}{\left(1-\kappa \right)}^{s-u-1}\eta^{T}\left(u\right)Q_{v}\eta \left(u\right)\}\\ = & \sum_{v=1}^{m}\{\left(\bar{b}-\underline{b}\right)\eta^{T}\left(s\right)Q_{v}\eta \left(s\right)+\sum_{n=s-\bar{b}+1}^{s-\underline{b}}\sum_{u=n+1}^{s-1}{\left(1-\kappa \right)}^{s-u}\eta^{T}\left(u\right)Q_{v}\eta \left(u\right)\\ \; & -\sum_{n=s-\bar{b}+1}^{s-\underline{b}}\sum_{u=n}^{s-1}{\left(1-\kappa \right)}^{s-u-1}\eta^{T}\left(u\right)Q_{v}\eta \left(u\right)\}\\ = & \sum_{v=1}^{m}\{\left(\bar{b}-\underline{b}\right)\eta^{T}\left(s\right)Q_{v}\eta \left(s\right)-\sum_{u=s-\bar{b}+1}^{s-\underline{b}}{\left(1-\kappa \right)}^{s-u}\eta^{T}\left(u\right)Q_{v}\eta \left(u\right)+\sum_{n=s-\bar{b}+1}^{s-\underline{b}}\sum_{u=n}^{s-1}{\left(1-\kappa \right)}^{s-u}\\ \; & \times \eta^{T}\left(u\right)Q_{v}\eta \left(u\right)-\sum_{n=s-\bar{b}+1}^{s-\underline{b}}\sum_{u=n}^{s-1}{\left(1-\kappa \right)}^{s-u-1}\eta^{T}\left(u\right)Q_{v}\eta \left(u\right)\}\\ = & \sum_{v=1}^{m}\{\left(\bar{b}-\underline{b}\right)\eta^{T}\left(s\right)Q_{v}\eta \left(s\right)-\sum_{u=s-\bar{b}+1}^{s-\underline{b}}{\left(1-\kappa \right)}^{s-u}\eta^{T}\left(u\right)Q_{v}\eta \left(u\right)\\ \; & -\kappa \sum_{n=s-\bar{b}+1}^{s-\underline{b}}\sum_{u=n}^{s-1}{\left(1-\kappa \right)}^{s-u-1}\eta^{T}\left(u\right)Q_{v}\eta \left(u\right)\}\\ = & \sum_{v=1}^{m}\left\{\left(\bar{b}-\underline{b}\right)\eta^{T}\left(s\right)Q_{v}\eta \left(s\right)-\sum_{u=s-\bar{b}+1}^{s-\underline{b}}{\left(1-\kappa \right)}^{s-u}\eta^{T}\left(u\right)Q_{v}\eta \left(u\right)\right\}-\kappa V_{3}\left(s,\eta \left(s\right)\right). \end{array}$$

Based on (3), we know that

(23) $$\begin{array}{c} {\left[\begin{array}{c} x_{i}\left(s\right)\\ g(x_{i}\left(s\right)) \end{array}\right]}^{T}\left[\begin{array}{cc} {\bar{\Xi }}_{1} & -{\bar{\Xi }}_{2}^{T}\\ {}* & I_{\left(l_{x}\right)} \end{array}\right]\left[\begin{array}{c} x_{i}\left(s\right)\\ g(x_{i}\left(s\right)) \end{array}\right]\leq 0, \end{array}$$

(24) $$\begin{array}{c} {\left[\begin{array}{c} e_{i}\left(s\right)\\ \bar{g}\left(e_{i}\left(s\right)\right) \end{array}\right]}^{T}\left[\begin{array}{cc} {\bar{\Xi }}_{1} & -{\bar{\Xi }}_{2}^{T}\\ {}* & I_{\left(l_{x}\right)} \end{array}\right]\left[\begin{array}{c} e_{i}\left(s\right)\\ \bar{g}\left(e_{i}\left(s\right)\right) \end{array}\right]\leq 0. \end{array}$$

The compact forms of (23) and (24) can be acquired as follows:

(25) $$\begin{array}{c} {\left[\begin{array}{c} \eta (s)\\ \overset{ˇ}{g}\left(\eta \left(s\right)\right) \end{array}\right]}^{T}\left[\begin{array}{cc} {\tilde{\Xi }}_{1} & -{\tilde{\Xi }}_{2}^{T}\\ {}* & I_{\left(2Ul_{x}\right)} \end{array}\right]\left[\begin{array}{c} \eta (s)\\ \overset{ˇ}{g}\left(\eta \left(s\right)\right) \end{array}\right]\leq 0. \end{array}$$

To summarize the above discussions, the following inequality is obtained:

(26) $$\begin{array}{cc} \; & \;\;\;E\left\{\Delta V(s,\eta (s),a)\right\}\leq \zeta^{T}\left(s\right)\Psi_{a}\zeta \left(s\right)-\kappa V\left(s,\eta \left(s\right),a\right)+\mathrm{tr}\left\{\Phi_{a}\right\} \end{array}$$

where

$$\begin{array}{cc} \zeta (s)≜ & {\left[\begin{array}{ccc} \eta^{T}\left(s\right) & \eta^{T}\left(s-b\left(s\right)\right) & {\overset{ˇ}{g}}^{T}\left(\eta \left(s\right)\right) \end{array}\right]}^{T},\\ \Phi_{a}≜ & \mathrm{diag}\{w_{0}^{2}F^{T}{\bar{P}}_{a}F,3v_{0}^{2}D^{T}I_{▵}^{T}\left(s\right)S_{a}^{T}{\bar{P}}_{a}S_{a}I_{▵}\left(s\right)D,3\xi_{0}^{2}I_{▵}^{T}\left(s\right)S_{a}^{T}{\bar{P}}_{a}S_{a}I_{▵}\left(s\right)\\ \; & +3h_{0}^{2}I_{▵}^{T}\left(s\right)S_{a}^{T}{\bar{P}}_{a}S_{a}I_{▵}\left(s\right)\}. \end{array}$$

Considering (15), we can obtain from (26) that

(27) $$\begin{array}{c} E\left\{V(s+1,\eta (s+1),\varkappa (s+1))|\eta (s),\varkappa (s)=a\right\}\leq \left(1-\kappa \right)E\{V\left(s,\eta \left(s\right),a\right)\}+\mathrm{tr}\left(\Phi_{a}\right). \end{array}$$

By utilizing Lemma 4, the following is obtained:

(28) $$\begin{array}{cc} \; & \;E\left\{V(s,\eta (s),a)\right\}\leq {\left(1-\kappa \right)}^{s}E\left\{V\left(0,\eta \left(0\right),a\right)\right\}+\frac{\left(1-{\left(1-\kappa \right)}^{s}\right)\mathrm{tr}\left(\Phi_{a}\right)}{\kappa }. \end{array}$$

Using (16), it can be observed that

(29) $$\begin{array}{c} E\left\{V(s,\eta (s),a)\right\}\geq \varphi_{\min}\left(P_{a}\right)E\left\{{∥\eta \left(s\right)∥}^{2}\right\} \end{array}$$

and

(30) $$\begin{array}{c} E\left\{V(0,\eta (0),a)\right\}\leq \bar{\phi }\underset{-\bar{b}\leq ȷ\leq 0}{\sup}{E\{∥\eta \left(ȷ\right)∥}^{2}\} \end{array}$$

where

$$\begin{array}{c} \bar{\phi }≜\varphi_{\max}\left(P_{a}\right)+\left({\bar{b}}^{2}-\bar{b}\underline{b}+\underline{b}\right)\sum_{v=1}^{m}\varphi_{\max}\left(Q_{v}\right) \end{array}$$

Accordingly, an inequality is obtained from (28)–(30) as follows:

$$\begin{array}{cc} E\left\{{∥\eta \left(s\right)∥}^{2}\right\}\leq & \frac{{\left(1-\kappa \right)}^{s}E\left\{V(0,\eta (0),a)\right\}}{\varphi_{\min}\left(P_{a}\right)}+\frac{\mathrm{tr}(\Phi )}{\kappa \varphi_{\min}\left(P_{a}\right)}\\ \leq & \frac{\bar{\phi }}{\varphi_{\min}\left(P_{a}\right)}{\left(1-\kappa \right)}^{s}\underset{-\bar{b}\leq ȷ\leq 0}{\sup}{E\{∥\eta \left(ȷ\right)∥}^{2}\}+\frac{\mathrm{tr}(\Phi )}{\kappa \varphi_{\min}\left(P_{a}\right)}. \end{array}$$

In terms of Definition 1, it can be observed that the dynamics of the estimation error are EUB in mean square, and the ultimate error bound is shown as follows:

$$\begin{array}{c} \frac{\mathrm{tr}(\Phi )}{\kappa \varphi_{\min}\left(P_{a}\right)}. \end{array}$$

The SE error can be expressed using e(s)≜Wη(s) with $W≜\left[\begin{array}{cc} 0_{\left(\left(Ul_{x}\right)\times \left(Ul_{x}\right)\right)} & I_{\left(Ul_{x}\right)} \end{array}\right]$. In the upcoming section, we aimed to design a gain parameter for a non-fragile estimator (8). □

**Theorem** **2.** *Consider that scalar 0<κ<1 is known. The constructed estimator (8) becomes an EUB state estimator of CN (1) in mean square as long as matrices $P_{a}≜\mathrm{diag}\left\{P_{1a},P_{2a}\right\}>0$ $(P_{1a}≜\mathrm{diag}\left\{P_{11a},P_{12a},\ldots ,P_{1Ua}\right\}$, $P_{2a}≜\mathrm{diag}\left\{P_{21a},P_{22a},\ldots ,P_{2Ua}\right\})$, $Q_{v}≜\mathrm{diag}\left\{Q_{1v},Q_{2v}\right\}>0$ $(Q_{1v}≜\mathrm{diag}\left\{Q_{11v},Q_{12v},\ldots ,Q_{1Uv}\right\}$, $Q_{2v}≜\mathrm{diag}\{Q_{21v},Q_{22v},\ldots ,$$Q_{2Uv}\}$, v=1,…,m), R>0 ($R=\mathrm{diag}\{R_{1},R_{2},R_{3}\}$), a matrix $G_{a}≜{\left[\begin{array}{cc} 0_{\left(\left(Ul_{y}\right)\times \left(Ul_{x}\right)\right)} & G_{2a}^{T} \end{array}\right]}^{T}$$(G_{2a}≜\mathrm{diag}\{G_{21a},G_{22a},\ldots ,$$G_{2Ua}\})$, and scalars $\lambda_{a}>0$, $\varepsilon_{a}>0$, and $\varrho_{a}>0$ satisfy the following linear matrix inequalities:*

(31) $$\begin{array}{c} \left[\begin{array}{ccccccc} \Upsilon_{11a} & * & * & * & * & * & *\\ 0 & \Upsilon_{22} & * & * & * & * & *\\ \Upsilon_{31a} & 0 & \Upsilon_{33a} & * & * & * & *\\ \Upsilon_{41a} & \Upsilon_{42a} & {\bar{P}}_{a}E & -{\bar{P}}_{a} & 0 & * & *\\ 0 & \Upsilon_{52a} & 0 & 0 & -I_{\left(m\right)}⊗{\bar{P}}_{a} & * & *\\ 0 & 0 & 0 & \Upsilon_{64a} & 0 & -\varepsilon_{a}I_{\left(2Ul_{y}\right)} & *\\ \Upsilon_{71a} & 0 & 0 & 0 & 0 & 0 & -\varepsilon_{a}I_{\left(2Ul_{y}\right)} \end{array}\right]<0, \end{array}$$

(32) $$\begin{array}{c} \left[\begin{array}{cccc} -R & * & * & *\\ K_{a} & -I_{\left(4\right)}⊗{\bar{P}}_{a} & * & *\\ 0 & {\hat{m}}_{a}^{T} & -\varrho_{a}I_{\left(2Ul_{y}\right)} & *\\ \varrho_{a}\hat{n} & 0 & 0 & -\varrho_{a}I_{\left(2Ul_{y}\right)} \end{array}\right]<0, \end{array}$$

(33) $$\begin{array}{c} W^{T}W<P_{a} \end{array}$$

*where*

$$\begin{array}{cc} \Upsilon_{11a} & ≜-\lambda_{a}{\tilde{\Xi }}_{1}+\left(1+\bar{b}-\underline{b}\right)\sum_{v=1}^{m}Q_{v}-P_{a},\;\Upsilon_{22}≜-{\left(1-\kappa \right)}^{\bar{b}}\bar{Q},\\ \Upsilon_{33a} & ≜-\lambda_{a}I_{\left(2Ul_{x}\right)},\;\Upsilon_{41a}≜{\bar{P}}_{a}{\bar{A}}_{a}+G_{a}{\bar{C}}_{1},\;\Upsilon_{42a}≜{\bar{P}}_{a}\left(\overset{ˇ}{\Theta }⊗B_{b2}\right),\\ \;\Upsilon_{52a} & ≜\left(I_{\left(m\right)}⊗{\bar{P}}_{a}\right)\hat{\Theta }\left(I_{\left(m\right)}⊗B_{b1}\right),\;\Upsilon_{31a}≜\lambda_{a}{\tilde{\Xi }}_{2},\;\Upsilon_{64a}≜-{\bar{m}}^{T}G_{a}^{T},\\ \Upsilon_{71a} & ≜\mathrm{diag}\left\{0_{\left(\left(Ul_{y}\right)\times \left(Ul_{x}\right)\right)},\varepsilon_{a}\left(I_{\left(U\right)}⊗n\right)C\right\},\;{\bar{C}}_{1}≜\left[\begin{array}{cc} 0_{\left(\left(Ul_{y}\right)\times \left(Ul_{x}\right)\right)} & -C \end{array}\right],\\ {\bar{A}}_{a} & ≜\mathrm{diag}\left\{A+\Lambda^{\left(a\right)}⊗\Gamma^{\left(a\right)},A+\Lambda^{\left(a\right)}⊗\Gamma^{\left(a\right)}\right\},\;\bar{m}≜\left[\begin{array}{cc} 0_{\left(\left(Ul_{y}\right)\times \left(Ul_{y}\right)\right)} & I_{\left(U\right)}⊗m \end{array}\right],\\ K_{a} & ≜\left[\begin{array}{ccc} w_{0}{\bar{P}}_{a}F & 0 & 0\\ 0 & \sqrt{3}v_{0}G_{a}D & 0\\ 0 & 0 & \sqrt{3}\xi_{0}G_{a}\\ 0 & 0 & \sqrt{3}h_{0}G_{a} \end{array}\right],\;\hat{n}≜\left[\begin{array}{ccc} 0 & (I_{\left(U\right)}⊗n)D & 0\\ 0 & 0 & I_{\left(U\right)}⊗n \end{array}\right],\\ {\hat{m}}_{a} & ≜\left[\begin{array}{cc} 0 & 0\\ \sqrt{3}v_{0}G_{a}\left(I_{\left(U\right)}⊗m\right) & 0\\ 0 & \sqrt{3}\xi_{0}G_{a}\left(I_{\left(U\right)}⊗m\right)\\ 0 & \sqrt{3}h_{0}G_{a}\left(I_{\left(U\right)}⊗m\right) \end{array}\right]. \end{array}$$

*Additionally, the minimum ultimate bound of estimation error $E\left\{{∥e(s)∥}^{2}\right\}$ is attained via settling the following optimization issue:*

(34) $$\begin{array}{c} \min_{\mathrm{subject}\;\mathrm{to}\;(31)–(33)}\mathrm{tr}\left(R\right). \end{array}$$

*The gain expression for the state estimator (8) is presented as follows:*

(35) $$\begin{array}{c} S_{ia}≜{\bar{P}}_{2ia}^{-1}G_{2ia}. \end{array}$$

**Proof** **of Theorem 2.** Defining ${\bar{P}}_{a}S_{a}≜G_{a}$ and ${\bar{P}}_{2ia}S_{ia}≜G_{2ia}$, through the application of Schur Complement Lemma and Lemma 5, (15) holds as long as (31) holds.

Considering $\Phi_{a}={\hat{K}}_{a}^{T}{\left(I_{\left(4\right)}⊗{\bar{P}}_{a}\right)}^{-1}{\hat{K}}_{a}$ with

$$\begin{array}{cc} {\hat{K}}_{a} & ≜\left[\begin{array}{ccc} w_{0}{\bar{P}}_{a}F & 0 & 0\\ 0 & \sqrt{3}v_{0}{\bar{P}}_{a}S_{a}I_{▵}\left(s\right)D & 0\\ 0 & 0 & \sqrt{3}\xi_{0}{\bar{P}}_{a}S_{a}I_{▵}\left(s\right)\\ 0 & 0 & \sqrt{3}h_{0}{\bar{P}}_{a}S_{a}I_{▵}\left(s\right) \end{array}\right], \end{array}$$

we can obtain from (32) that

(36) $$\begin{array}{c} \Phi_{a}<R. \end{array}$$

By combining (28) with (36), we can derive the following:

(37) $$\begin{array}{cc} E\left\{V(s,\eta (s),a)\right\} & <{\left(1-\kappa \right)}^{s}E\{V\left(0,\eta \left(0\right),a\right)\}\;+\;\frac{\mathrm{tr}(\Phi_{a})}{\kappa }\\ \; & <{\left(1-\kappa \right)}^{s}E\{V\left(0,\eta \left(0\right),a\right)\}\;+\;\frac{\mathrm{tr}(R)}{\kappa }. \end{array}$$

Based on (33) and (30), we can derive the following:

(38) $$\begin{array}{cc} \;E\left\{{∥e(s)∥}^{2}\right\}\; & =E\left\{\eta^{T}\left(s\right)W^{T}W\eta \left(s\right)\right\}\\ \; & <E\left\{\eta^{T}\left(s\right)P_{a}\eta \left(s\right)\right\}\\ \; & <E\left\{V(s,\eta (s),a)\right\}\\ \; & <{\left(1-\kappa \right)}^{s}E\left\{V(0,\eta (0),a)\right\}+\frac{\mathrm{tr}(R)}{\kappa }\\ \; & <\bar{\phi }{\left(1-\kappa \right)}^{s}\underset{-\bar{b}\leq ȷ\leq 0}{\sup}{E\{∥\eta \left(ȷ\right)∥}^{2}\}\;+\;\frac{\mathrm{tr}(R)}{\kappa }. \end{array}$$

By considering (38), $\frac{\mathrm{tr}(R)}{\kappa }$ is the ultimate bound of $E\left\{{∥e(s)∥}^{2}\right\}$. Through minimizing tr(R), the minimum of such an ultimate bound is attained, which ends the proof. □

Note that the result yielded in Theorem 2 can be generalized to more ordinary situations, e.g., the SE of CNs with a constant time delay. Change $\sum_{l=1}^{m}{϶}_{il}\left(s\right)x_{i}\left(s-b_{l}\left(s\right)\right)$ to $x_{i}\left(s-b\right)$ with a given scalar b>0. The following corollary was obtained from Theorem 2, which was used to design a state estimator that satisfies the exponential ultimate boundedness in mean square for CN (1) with a constant time delay.

**Corollary** **1.** *Let scalar 0<κ<1 be known. The constructed estimator (8) becomes an EUB state estimator of CN (1) in mean square as long as matrices $P_{a}≜\mathrm{diag}\left\{P_{1a},P_{2a}\right\}>0$ $(P_{1a}≜\mathrm{diag}\{$$P_{11a},P_{12a},\ldots ,P_{1Ua}$}, $P_{2a}≜\mathrm{diag}\left\{P_{21a},P_{22a},\ldots ,P_{2Ua}\right\})$, $Q≜\mathrm{diag}\{Q_{1},Q_{2}\}>0$ $(Q_{1}≜\mathrm{diag}\left\{Q_{11},Q_{12},\ldots ,Q_{1U}\right\}$, $Q_{2}≜\mathrm{diag}\left\{Q_{21},Q_{22},\ldots ,Q_{2U}\right\})$, R>0 ($R=\mathrm{diag}\{R_{1},R_{2},R_{3}\}$), a matrix $G_{a}≜{\left[\begin{array}{cc} 0_{\left(\left(Ul_{y}\right)\times \left(Ul_{x}\right)\right)} & G_{2a}^{T} \end{array}\right]}^{T}$$(G_{2a}≜\mathrm{diag}\{G_{21a},G_{22a},$$\ldots ,G_{2Ua}\})$, and scalars $\lambda_{a}>0$, $\varepsilon_{a}>0$, and $\varrho_{a}>0$ satisfy (32), (33) and the following linear matrix inequality:*

(39) $$\begin{array}{c} \left[\begin{array}{cccccc} {\bar{\Upsilon }}_{11a} & * & * & * & * & *\\ 0 & {\bar{\Upsilon }}_{22} & * & * & * & *\\ \Upsilon_{31a} & 0 & \Upsilon_{33a} & * & * & *\\ \Upsilon_{41a} & {\bar{\Upsilon }}_{42a} & {\bar{P}}_{a}E & -{\bar{P}}_{a} & * & *\\ 0 & 0 & 0 & \Upsilon_{64a} & -\varepsilon_{a}I_{\left(2Ul_{y}\right)} & *\\ \Upsilon_{71a} & 0 & 0 & 0 & 0 & -\varepsilon_{a}I_{\left(2Ul_{y}\right)} \end{array}\right]<0 \end{array}$$

*where*

$$\begin{array}{cc} \; & {\bar{\Upsilon }}_{11a}≜-\lambda {\tilde{\Xi }}_{1}+Q-P_{a},\;{\bar{\Upsilon }}_{22}≜-Q,\;{\bar{\Upsilon }}_{42a}≜{\bar{P}}_{a}B_{b2}. \end{array}$$

Additionally, the minimum value of the ultimate bound of $E\left\{{∥e(s)∥}^{2}\right\}$ was determined via tackling the following optimization issue:

(40) $$\begin{array}{c} \min_{\mathrm{subject}\;\mathrm{to}\;(32),\;(33)\;\mathrm{and}\;(39)}\mathrm{tr}\left(R\right). \end{array}$$

The gain parameter is represented as (35) of the non-fragile estimator (8).

**Remark** **4.** *In this paper, the issue of non-fragile SE is discussed, focusing on nonlinear delayed CNs subject to random couplings using BESs. The major advantages presented by this paper compared to existing SE approaches for CNs are as follows: (1) the issue of non-fragile SE is considered for the first time for nonlinear CNs with multiple stochastic communication time delays and random couplings under BESs; (2) the impact of the phenomenon of random bit errors considered regarding the capability of the state estimator; and (3) the proposed SE approach ensures that the SE error dynamics are EUB in mean square and the minimum ultimate bound is obtained.*

In the subsequent section, two confirmatory examples are used to verify the rationality and effectiveness of the obtained SE method.

## 4. Simulation Examples

**Example** **1.** *Consider a CN (1) (* U=5 *) with the following parameters:*

$$\begin{array}{cc} \; & p=2,\;M=6,\;q=0.01,\;\rho =0.0355,\;w_{0}=0.02,\;v_{0}=0.04,\;m=2,\;\bar{b}=4,\;\underline{b}=1,\\ \; & {\bar{϶}}_{11}={\bar{϶}}_{21}={\bar{϶}}_{31}={\bar{϶}}_{41}={\bar{϶}}_{51}=0.8,\;{\bar{϶}}_{12}={\bar{϶}}_{22}={\bar{϶}}_{32}={\bar{϶}}_{42}={\bar{϶}}_{52}=0.6,\;\rho =0.0355,\\ \; & \pi_{11}=0.55,\;\pi_{12}=0.45,\;\pi_{21}=0.4,\;\pi_{22}=0.6,\;D_{1}=D_{2}=0.03,\;D_{3}=D_{4}=0.02,\\ \; & \Gamma^{\left(1\right)}=\mathrm{diag}\left\{0.52,0.53,0.52\right\},\;\Gamma^{\left(2\right)}=\mathrm{diag}\left\{0.64,0.63,0.65\right\},\;m=1,\;n=0.3,\;D_{5}=0.01,\\ \; & \Lambda^{\left(1\right)}=\left[\begin{array}{ccccc} -0.4 & 0.15 & 0.08 & 0.12 & 0.05\\ 0.15 & -0.4 & 0.1 & 0.07 & 0.08\\ 0.08 & 0.1 & -0.3 & 0.04 & 0.08\\ 0.12 & 0.07 & 0.04 & -0.3 & 0.07\\ 0.05 & 0.08 & 0.08 & 0.07 & -0.28 \end{array}\right],\;\Lambda^{\left(2\right)}=\left[\begin{array}{ccccc} -0.2 & 0.05 & 0.05 & 0.05 & 0.05\\ 0.05 & -0.2 & 0.05 & 0.05 & 0.05\\ 0.05 & 0.05 & -0.2 & 0.05 & 0.05\\ 0.05 & 0.05 & 0.05 & -0.2 & 0.05\\ 0.05 & 0.05 & 0.05 & 0.05 & -0.2 \end{array}\right],\\ \; & A_{1}=A_{2}=\left[\begin{array}{ccc} 0.01 & 0 & 0.009\\ 0 & 0.001 & 0.022\\ 0.011 & 0.012 & 0.018 \end{array}\right],\;A_{3}=A_{4}=\left[\begin{array}{ccc} -0.02 & -0.02 & -0.01\\ 0.01 & 0.03 & -0.01\\ 0.03 & 0.02 & -0.01 \end{array}\right],\;\kappa =0.01,\\ \; & A_{5}=\left[\begin{array}{ccc} -0.02 & -0.01 & -0.02\\ 0.01 & 0.02 & -0.03\\ -0.01 & 0.04 & -0.02 \end{array}\right],B_{b1}=B_{b2}=\left[\begin{array}{ccc} 0.02 & -0.03 & 0.01\\ 0.04 & 0.01 & 0.02\\ 0.02 & -0.01 & 0.03 \end{array}\right],\\ \; & B_{b3}=B_{b4}=\left[\begin{array}{ccc} 0.04 & -0.01 & 0.03\\ 0.02 & 0.02 & 0.02\\ 0.01 & -0.02 & 0.02 \end{array}\right],\;B_{b5}=\left[\begin{array}{ccc} 0.02 & -0.01 & 0.02\\ 0.02 & 0.03 & 0.02\\ 0.02 & -0.02 & 0.04 \end{array}\right],\\ \; & C_{1}=C_{2}=\left[\begin{array}{ccc} -0.1 & 0.2 & -0.1 \end{array}\right],\;C_{3}=C_{4}=\left[\begin{array}{ccc} -0.1 & 0.2 & -0.1 \end{array}\right],\\ \; & C_{5}=\left[\begin{array}{ccc} -0.1 & 0.2 & -0.1 \end{array}\right],\;E_{1}=E_{2}=E_{3}=E_{4}=E_{5}=\left[\begin{array}{ccc} 1 & 1 & 1\\ 1 & 1 & 1\\ 1 & 1 & 1 \end{array}\right],\\ \; & F_{1}=F_{2}=\left[\begin{array}{c} 0.01\\ 0.01\\ 0 \end{array}\right],F_{3}=F_{4}=\left[\begin{array}{c} -0.01\\ -0.01\\ -0.01 \end{array}\right],F_{5}=\left[\begin{array}{c} -0.02\\ -0.01\\ -0.02 \end{array}\right]. \end{array}$$

*The nonlinear function *$g(x_{i}\left(s\right))$ (i=1,2,…,U)* is chosen as follows:*

$$\begin{array}{c} g\left(x_{i}\left(s\right)\right)=\left[\begin{array}{c} -0.06x_{i1}\left(s\right)+0.03x_{i2}\left(s\right)+\tanh\left(0.03x_{i1}\left(s\right)\right)\\ 0.06x_{i2}\left(s\right)-\tanh\left(0.02x_{i2}\left(s\right)\right)\\ -0.06x_{i2}\left(s\right)+0.03x_{i3}\left(s\right)+\tanh\left(0.03x_{i2}\left(s\right)\right) \end{array}\right] \end{array}$$

*where* $x_{i\sigma }\left(s\right)\left(\sigma =1,2,3\right)$* is the *σ*th element of *$x_{i}\left(s\right)$*. It can be observed that (3) is fulfilled as follows:*

$$\begin{array}{cc} \Xi_{1} & =\left[\begin{array}{ccc} -0.06 & 0.03 & 0\\ 0 & 0.04 & 0\\ 0 & -0.06 & 0.03 \end{array}\right]\mathrm{and}\Xi_{2}=\left[\begin{array}{ccc} -0.03 & 0.03 & 0\\ 0 & 0.06 & 0\\ 0 & -0.03 & 0.03 \end{array}\right]. \end{array}$$

*By solving the optimization issue (34) subject to matrix inequalities (31)–(33), the gain parameters of the estimator (8) are calculated as follows:*

$$\begin{array}{cc} S_{11} & =10^{-14}\times \left[\begin{array}{c} 0.1015\\ 0.0261\\ 0.2212 \end{array}\right],\;S_{21}=10^{-15}\times \left[\begin{array}{c} 0.2787\\ -0.4131\\ 0.2407 \end{array}\right],\;S_{31}=10^{-16}\times \left[\begin{array}{c} 0.7013\\ -0.7124\\ 0.0248 \end{array}\right],\\ S_{41} & =10^{-17}\times \left[\begin{array}{c} 0.2144\\ -0.2898\\ 0.1688 \end{array}\right],S_{51}=10^{-17}\times \left[\begin{array}{c} 0.0952\\ -0.1899\\ 0.0227 \end{array}\right],\;S_{12}=10^{-15}\times \left[\begin{array}{c} 0.1812\\ 0.0621\\ 0.1680 \end{array}\right],\\ S_{22} & =10^{-18}\times \left[\begin{array}{c} 0.6679\\ -0.8591\\ 0.7532 \end{array}\right],\;S_{32}=10^{-18}\times \left[\begin{array}{c} 0.2586\\ -0.4815\\ 0.4934 \end{array}\right],S_{42}=10^{-16}\times \left[\begin{array}{c} -0.0004\\ 0.0001\\ 0.1626 \end{array}\right],\\ S_{52} & =10^{-13}\times \left[\begin{array}{c} 0.0879\\ -0.5279\\ 0.1690 \end{array}\right]. \end{array}$$

**Figure 3:**
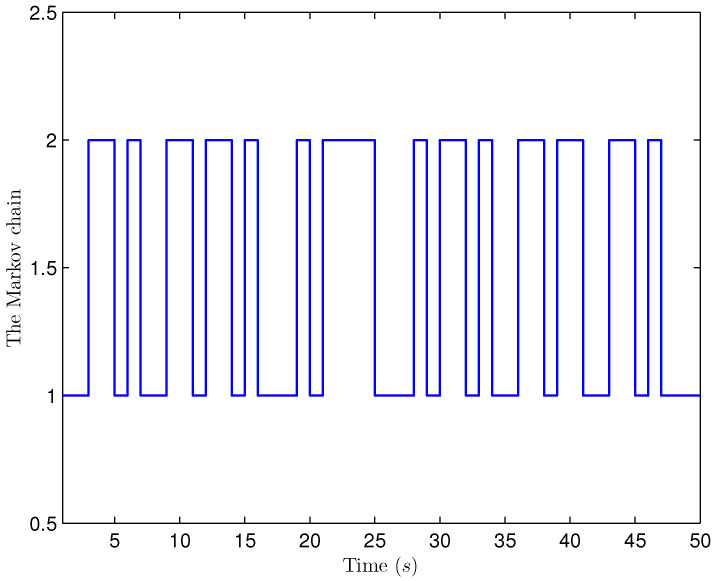
Mode evolution.

**Figure 4:**
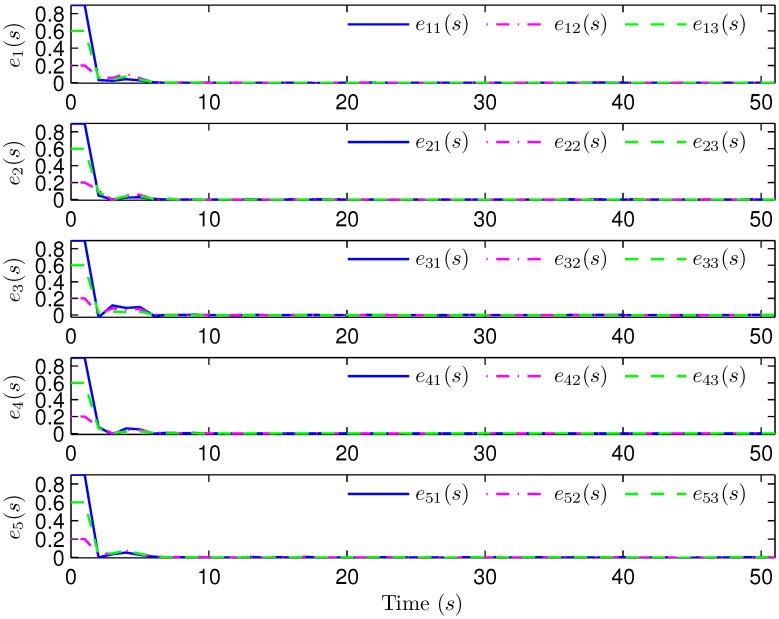
State estimation error of five nodes.

**Figure 5:**
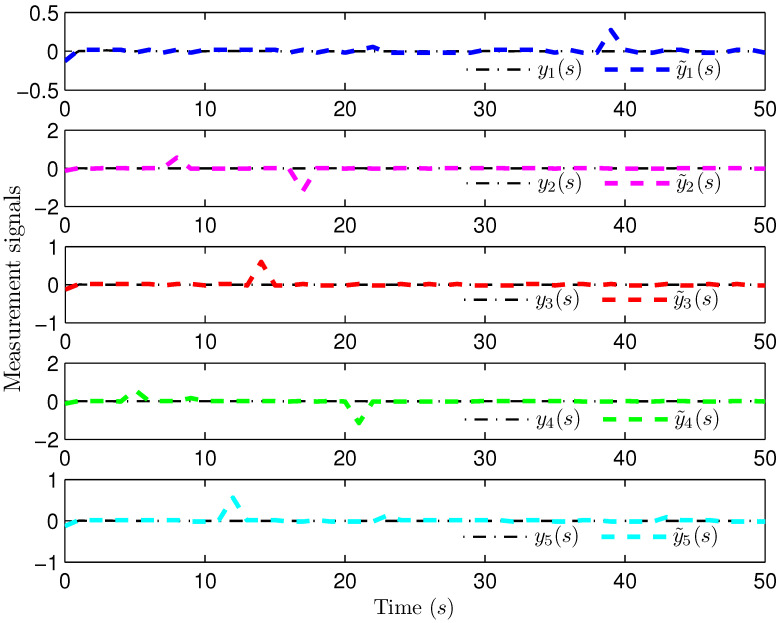
The ideal and received measurement outputs.

**Figure 6:**
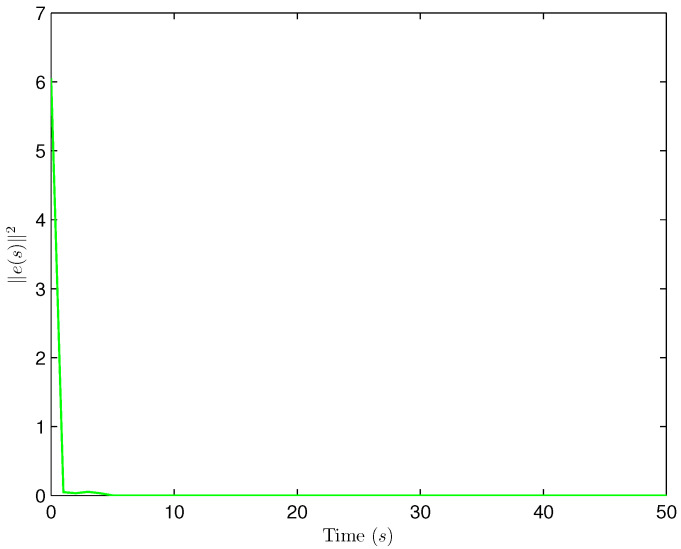
The norm of the estimation error e(s).

**Figure 7:**
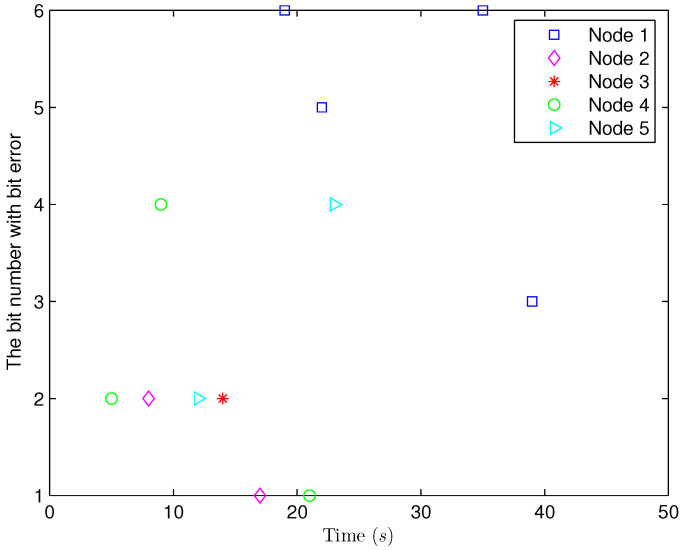
The occurrence of random bit errors.

*Select the initial conditions *$x_{i}\left(\varsigma \right)={\left[\begin{array}{ccc} 0.9 & 0.2 & 0.6 \end{array}\right]}^{T}$* and *${\hat{x}}_{i}\left(\varsigma \right)={\left[\begin{array}{ccc} 0 & 0 & 0 \end{array}\right]}^{T}$ (ς=−4,−3,…,0,i=1,2,…,5)*. Select *H(s)=cos(3s+1)* and *$b_{1}\left(s\right)=b_{2}\left(s\right)=3+{\left(-1\right)}^{s}$*. The simulation figures are presented in Figure 3, Figure 4, Figure 5, Figure 6 and Figure 7. Figure 3 shows the current mode describing randomly varying couplings, where “1” and “2” in the vertical coordinate represent the first and second mode of couplings, respectively. In Figure 4, the estimation error is represented by five nodes. Figure 4 shows that the estimation error for each node is low and gradually approximates to 0, which illustrates that the SE is exact. The original measurement signal and the one received by the estimator are plotted in Figure 5, and the deviation between them indicates the existence of random bit errors. Figure 6 depicts the norm of the estimation error (i.e., *${∥e(s)∥}^{2}$*). From Figure 6, we see that *${∥e(s)∥}^{2}$* is bounded; that is, the estimator performance requirement (14) of exponential ultimate boundedness in mean square is fulfilled for CN (1). In Figure 7, the occurrence of random bit errors is revealed. For node 2, bit flipping occurs in the second bit and the first bit at time steps 8 and 17, respectively, which causes signal distortion at the same time steps in Figure 5. Furthermore, the higher bit undergoes bit flipping, and a larger deviation appears between the original measurement outputs and the received ones. In summary, the estimation results obtained by the designed non-fragile estimator are good.*

**Figure 8:**
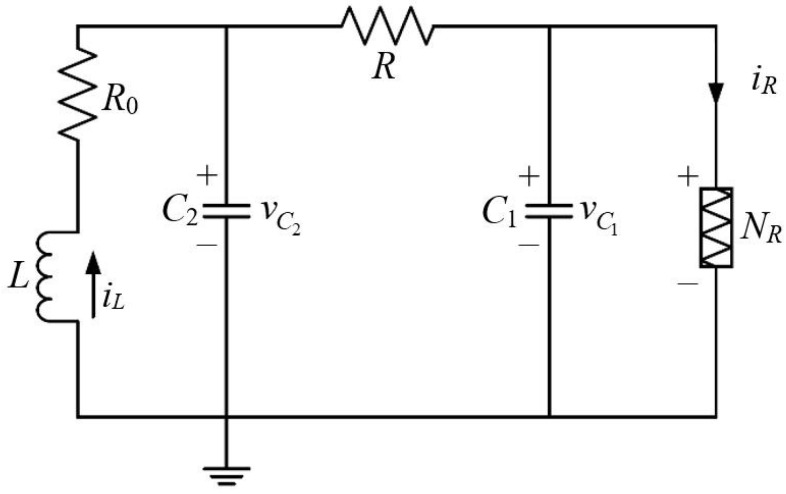
Chua’s circuit.

**Example** **2.** *For a delayed complex network (1) (*U=4*) whose local dynamical system (*$l_{x}=3$*) is a discrete-time Chua’s circuit (as is depicted in Figure 8), *$x_{1}\left(s\right)$, $x_{2}\left(s\right)$* and *$x_{3}\left(s\right)$* denote voltages *$v_{C_{1}}$* and *$v_{C_{2}}$* across the linear capacitors with capacitances *$C_{1}$* and *$C_{2}$*, and the current *$i_{L}$* passing the linear inductor L, respectively; and the nonlinear function *g(·)* describes voltage versus the current feature of the nonlinear resistor *$N_{R}$* (Chua’s diode) [5,69].*

*The parameters of such a complex network are presented as follows:*

$$\begin{array}{cc} \; & \kappa =0.01,\pi_{11}=0.65,\pi_{12}=0.35,\pi_{21}=0.5,\pi_{22}=0.5,w_{0}=0.02,v_{0}=0.04,m=2,\\ \; & \Lambda^{\left(1\right)}=\left[\begin{array}{cccc} -0.4 & 0.15 & 0.15 & 0.1\\ 0.2 & -0.6 & 0.2 & 0.2\\ 0.15 & 0.15 & -0.5 & 0.2\\ 0.2 & 0.2 & 0.2 & -0.6 \end{array}\right],\Lambda^{\left(2\right)}=\left[\begin{array}{cccc} -0.64 & 0.22 & 0.22 & 0.2\\ 0.22 & -0.58 & 0.12 & 0.24\\ 0.22 & 0.12 & -0.44 & 0.1\\ 0.2 & 0.24 & 0.1 & -0.54 \end{array}\right],\\ \; & A_{1}=\left[\begin{array}{ccc} 0.6 & 0.4 & 0\\ 0.3 & 0.7 & 0.2\\ 0 & -5.4 & 0.8 \end{array}\right],A_{2}=A_{1},A_{3}=\left[\begin{array}{ccc} 0.8 & 0.2 & 0\\ 0.25 & 0.75 & 0.1\\ 0 & -0.2 & 0.9 \end{array}\right],A_{4}=A_{3},\\ \; & B_{b1}=B_{b2}=B_{b3}=B_{b4}=0.01I_{\left(3\right)},\rho =0.0355,{\bar{϶}}_{11}={\bar{϶}}_{21}={\bar{϶}}_{31}={\bar{϶}}_{41}=0.4,\\ \; & \Gamma^{\left(1\right)}=I_{\left(3\right)},\Gamma^{\left(2\right)}=0.5I_{\left(3\right)},q=0.01,m=I_{\left(2\right)},{\bar{϶}}_{12}={\bar{϶}}_{22}={\bar{϶}}_{32}={\bar{϶}}_{42}=0.5,M=6,\\ \; & C_{1}=C_{2}=C_{3}=C_{4}=\left[\begin{array}{ccc} 1 & 0 & 0\\ 0 & 1 & 0 \end{array}\right],D_{1}=\left[\begin{array}{c} 0.003\\ 0.003 \end{array}\right],D_{3}=\left[\begin{array}{c} 0.002\\ 0.002 \end{array}\right],n=\left[\begin{array}{cc} 0.01 & 0.01\\ 0.01 & 0.01 \end{array}\right],\\ \; & D_{2}=D_{1},D_{4}=D_{3},E_{1}=E_{2}=E_{3}=E_{4}=I_{\left(3\right)},F_{1}=F_{2}=F_{3}=F_{4}=I_{\left(3\right)},\underline{b}=1,\bar{b}=4. \end{array}$$

*The expression of nonlinear function* $g(x_{i}\left(s\right))$ *(* i=1,2,…,4 *) is*

$$g\left(x_{i}\left(s\right)\right)=\left[\begin{array}{c} -0.2x_{i1}\left(s\right)+0.3(|x_{i1}\left(s\right)+2|-|x_{i1}\left(s\right)-2\left|\right)\\ 0\\ 0 \end{array}\right],$$

*and it was observed that* $\Xi_{1}=\mathrm{diag}\left\{-0.8,0,0\right\}$ *and* $\Xi_{2}=\mathrm{diag}\left\{-0.2,0,0\right\}$ *satisfy (3).*

*By solving the optimization issue (34), the gain parameters of estimator (8) are computed as follows:*

$$\begin{array}{cc} \; & S_{11}=10^{-7}\times \left[\begin{array}{cc} 0.0098 & -0.0376\\ 0.0292 & -0.1120\\ 0.0125 & -0.0480 \end{array}\right],S_{21}=10^{-8}\times \left[\begin{array}{cc} 0.0064 & 0.0365\\ 0.0182 & 0.1017\\ 0.0032 & -0.0254 \end{array}\right],\\ \; & S_{31}=10^{-12}\times \left[\begin{array}{cc} 0.1480 & -0.2986\\ -0.1049 & -0.2305\\ -0.0446 & -0.0712 \end{array}\right],S_{41}=10^{-12}\times \left[\begin{array}{cc} 0.0064 & -0.0917\\ -0.1303 & -0.1586\\ -0.0650 & 0.0492 \end{array}\right],\\ \; & S_{12}=10^{-9}\times \left[\begin{array}{cc} 0.9808 & 0.2382\\ -0.0243 & -0.0038\\ -0.0124 & -0.0011 \end{array}\right],S_{22}=10^{-12}\times \left[\begin{array}{cc} -0.3878 & 0.6634\\ -0.2159 & 0.5081\\ -0.2846 & 0.7688 \end{array}\right],\\ \; & S_{32}=10^{-12}\times \left[\begin{array}{cc} -0.4269 & 0.0116\\ -0.0791 & -0.0603\\ -0.2546 & 0.0351 \end{array}\right],S_{42}=10^{-9}\times \left[\begin{array}{cc} 0.0189 & 0.0205\\ 0.1827 & 0.1960\\ 0.0793 & 0.0852 \end{array}\right]. \end{array}$$

**Figure 9:**
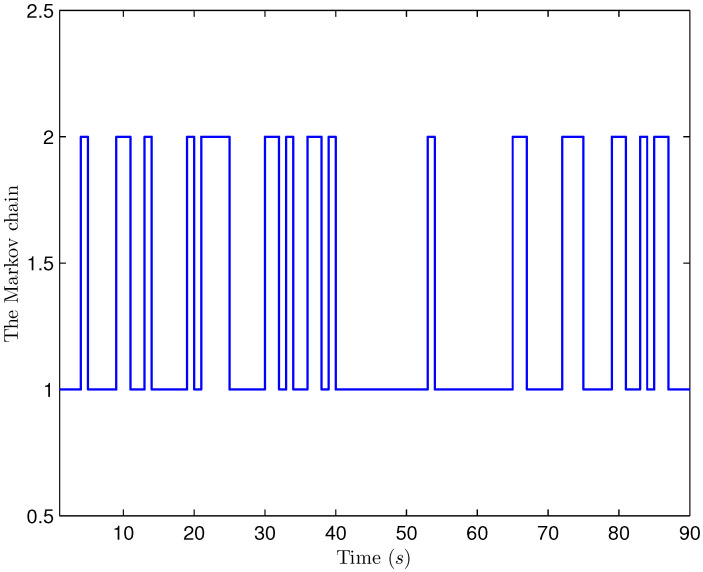
Mode evolution.

**Figure 10:**
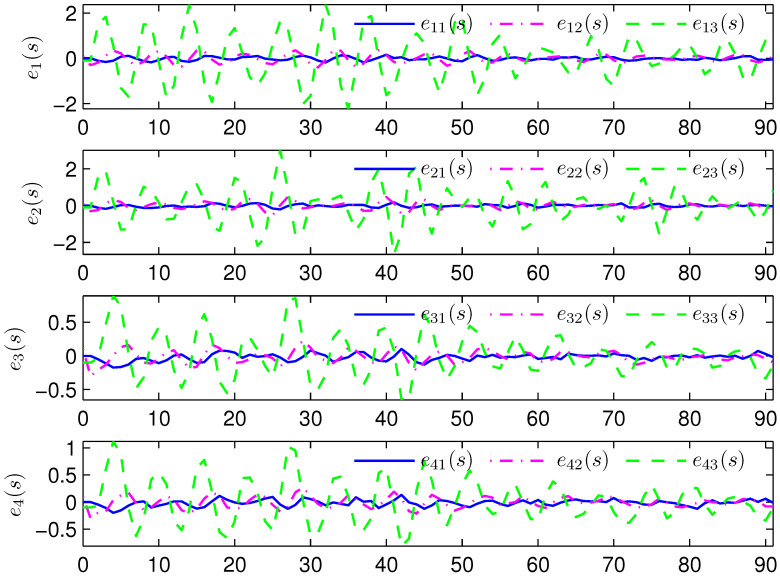
State estimation error of four nodes.

**Figure 11:**
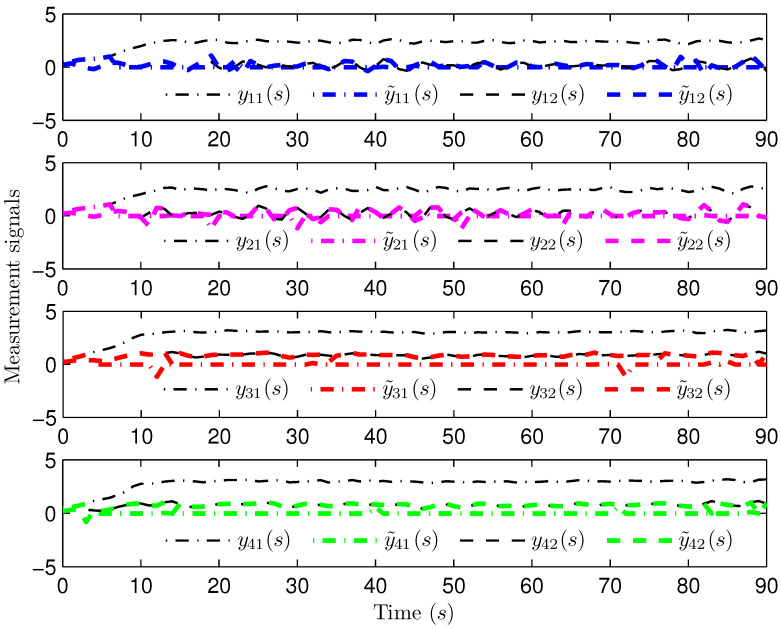
The measurement output and the estimator input.

**Figure 12:**
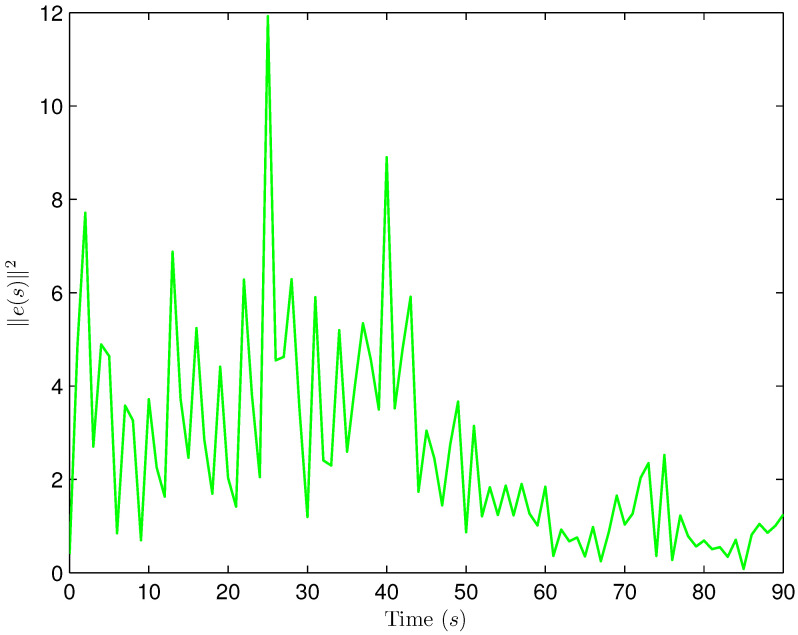
The norm of the estimation error e(s).

**Figure 13:**
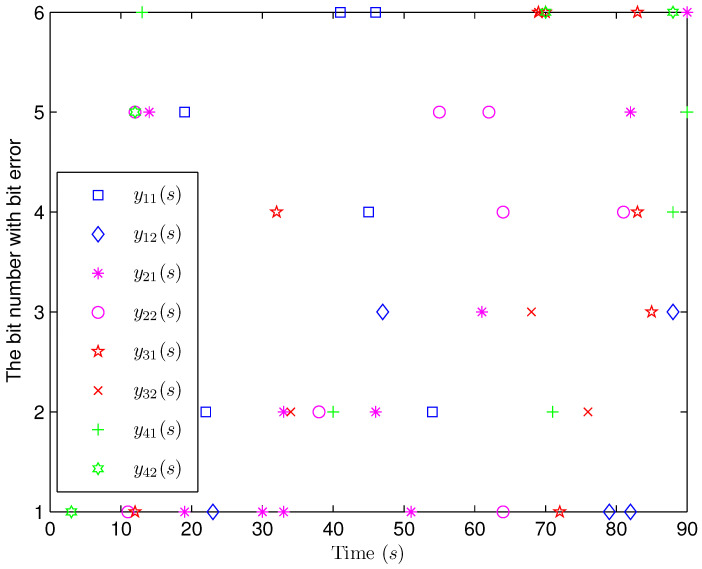
The occurrence of random bit errors.

*The initial states are given as *$x_{i}\left(-4\right)={\left[\begin{array}{ccc} 0.9 & 0.2 & 0.6 \end{array}\right]}^{T}$, $x_{i}\left(-3\right)={\left[\begin{array}{ccc} 0.8 & 0.4 & 0.2 \end{array}\right]}^{T}$, $x_{i}\left(-2\right)={\left[\begin{array}{ccc} 0.2 & 0.7 & 0.7 \end{array}\right]}^{T}$, $x_{i}\left(-1\right)={\left[\begin{array}{ccc} 0.3 & 0.4 & 0.2 \end{array}\right]}^{T}$, $x_{i}\left(0\right)={\left[\begin{array}{ccc} 0.4 & 0.2 & 0.1 \end{array}\right]}^{T}$, ${\hat{x}}_{i}\left(-4\right)={\left[\begin{array}{ccc} 0.3 & 0.4 & 0.1 \end{array}\right]}^{T}$, ${\hat{x}}_{i}\left(-3\right)={\left[\begin{array}{ccc} 0.5 & 0.4 & 0.2 \end{array}\right]}^{T}$, ${\hat{x}}_{i}\left(-2\right)={\left[\begin{array}{ccc} 0.1 & 0.6 & 0.5 \end{array}\right]}^{T}$, ${\hat{x}}_{i}\left(-1\right)={\left[\begin{array}{ccc} 0.3 & 0.3 & 0.3 \end{array}\right]}^{T}$* and *${\hat{x}}_{i}\left(0\right)={\left[\begin{array}{ccc} 0.4 & 0.5 & 0.2 \end{array}\right]}^{T}$* (*i=1,2,…,4). $H\left(s\right)=0.01\cos\left(3s+1\right)I_{\left(2\right)}$* and *$b_{1}\left(s\right)=b_{2}\left(s\right)=3+{\left(-1\right)}^{s}$* are chosen. Simulation curves are presented in Figure 9, Figure 10, Figure 11, Figure 12 and Figure 13. In Figure 9, the situation of mode switching is exhibited for the Markov chain, where “1” (or “2”) in the vertical coordinate indicates that coupling occurred with mode 1 (or mode 2). In Figure 10, the SE error *$e_{i}\left(s\right)$ (i=1,2,…,4*) is presented, which is within the interval *[−2.645,3.012]*. In Figure 11, the ideal measurement signal and the actually received one are shown, and there is a difference between them. When an accurate description of the received measurement signal is provided, instead of using the ideal measurement signal, the estimation performance is guaranteed. In Figure 12, the norm of the SE error *e(s)* is presented, which is bounded (not more than *11.92*). In Figure 13, for the BBS from measurement *$y_{i♭}$ (i=1,2,…,4, ♭=1,2*), bits that undergo flipping are presented. For the measurement component *$y_{11}\left(s\right)$*, bit flipping occurred in bits 5, 2, 6, 4, 6 and 2 at time steps 19, 22, 41, 45, 46 and 54, respectively. In light of the aforementioned outcomes, the developed non-fragile SE means presented in this paper are valid.*

## 5. Conclusions

The issue of non-fragile SE was investigated in this paper for a type of nonlinear delayed CN with randomly varying couplings under BESs. A Markov chain was used to reflect the stochastic variations in both the network topology and the inner coupling, in combination with the Kronecker delta function. The BESs were applied to the signal transmission between the sensor and the remote state estimator. The phenomenon of random bit errors was considered and described using a sequence of Bernoulli-distributed random variables. Considering the gain perturbations that occurred during the practical execution of the estimator, the non-fragile state estimator was modelled under different gain variations, which ensured that the SE error dynamics were EUB in mean square and the ultimate bound was minimized. The gain parameter of the established estimator was obtained by tackling the optimization problem constrained by some linear matrix inequalities. The working ability of the designed estimator was demonstrated through two expository examples. In future, we would extend the acquired result to the SE of (1) CNs using the available measurement outputs from only a portion of the network nodes [70,71]; (2) sensor networks [72,73]; and (3) other systems [74,75].

## Data Availability

No new data were created or analyzed in this study. Data sharing is not applicable to this article.

## Abbreviations

The following abbreviations are used in this manuscript:

CNs	Complex networks.
SE	State estimation.
NCSs	Networked control systems.
BES	Binary encoding scheme.
BBS	Binary bit string.
BSC	Binary symmetric channel.
BER	Bit error rate.
EUB	Exponentially ultimately bounded.
